# Supplementary material for: Strong microwave squeezing above 1 Tesla and 1 Kelvin
Source: Nat Commun. 2024 May 18;15:4229. doi: 10.1038/s41467-024-48519-3 (PMC11102506; doi:10.1038/s41467-024-48519-3)
Supplement: Supplementary file 1 — Supplementary Information [file 41467_2024_48519_MOESM1_ESM.pdf]

Supplementary Information: Strong Microwave Squeezing Above 1 Tesla and 1 Kelvin

Arjen Vaartjes,[1] Anders Kringhøj,[1] Wyatt Vine,[1] Tom Day,[1] Andrea Morello,[1] and Jarryd J. Pla[1,*]

[1]*School of Electrical Engineering and Telecommunications, UNSW Sydney, Sydney, NSW 2052, Australia*

(Dated: April 26, 2024)

CONTENTS

1. Supplementary Note 1: Experimental Setup 3
  A. Dilution Refrigerator Wiring 3
  B. Circulator Insertion Loss 4
  C. Thermal Noise Source 5

2. Supplementary Note 2: Theory 5
  A. Input-Output Theory 5
    1. Single KIPA in Phase-Sensitive Mode 5
    2. Input-Output Relation for the Loss Between Components 7
    3. Input-Output Relation for the Full Experimental Setup 7
  B. Limitations of the Direct Squeezing Measurements 8
  C. Amplifier Noise Calibration 9
  D. Squeezing of Thermal States 10

3. Supplementary Note 3: Device Fabrication, Design, and Packaging 11
  A. Fabrication 11
  B. Design 11
  C. Packaging 13

4. Supplementary Note 4: Characterization of the KIPAs 13
  A. Reflection Measurement 13
  B. Frequency Tunability 13
  C. Gain 14
  D. Gain-Bandwidth Product 14
  E. Independent Vacuum Squeezing 15
  F. 1-dB Compression Point 15
  G. Frequency Dependence of Pump Transmission 16
  H. Variation of Resonance Frequency and Quality Factors with Magnetic Field 17
  I. Variation of Resonance Frequency and Quality Factor with Temperature 17
  J. Amplifier Added Noise 17

5. Supplementary Note 5: Pump Crosstalk 19
  A. Influence of the SQZ pump on the AMP Gain 19
  B. Influence of the SQZ Pump on the AMP Amplified Noise and Vice Versa 20

6. Supplementary Note 6: Supplementary Figures 21

References 24

* jarryd@unsw.edu.au

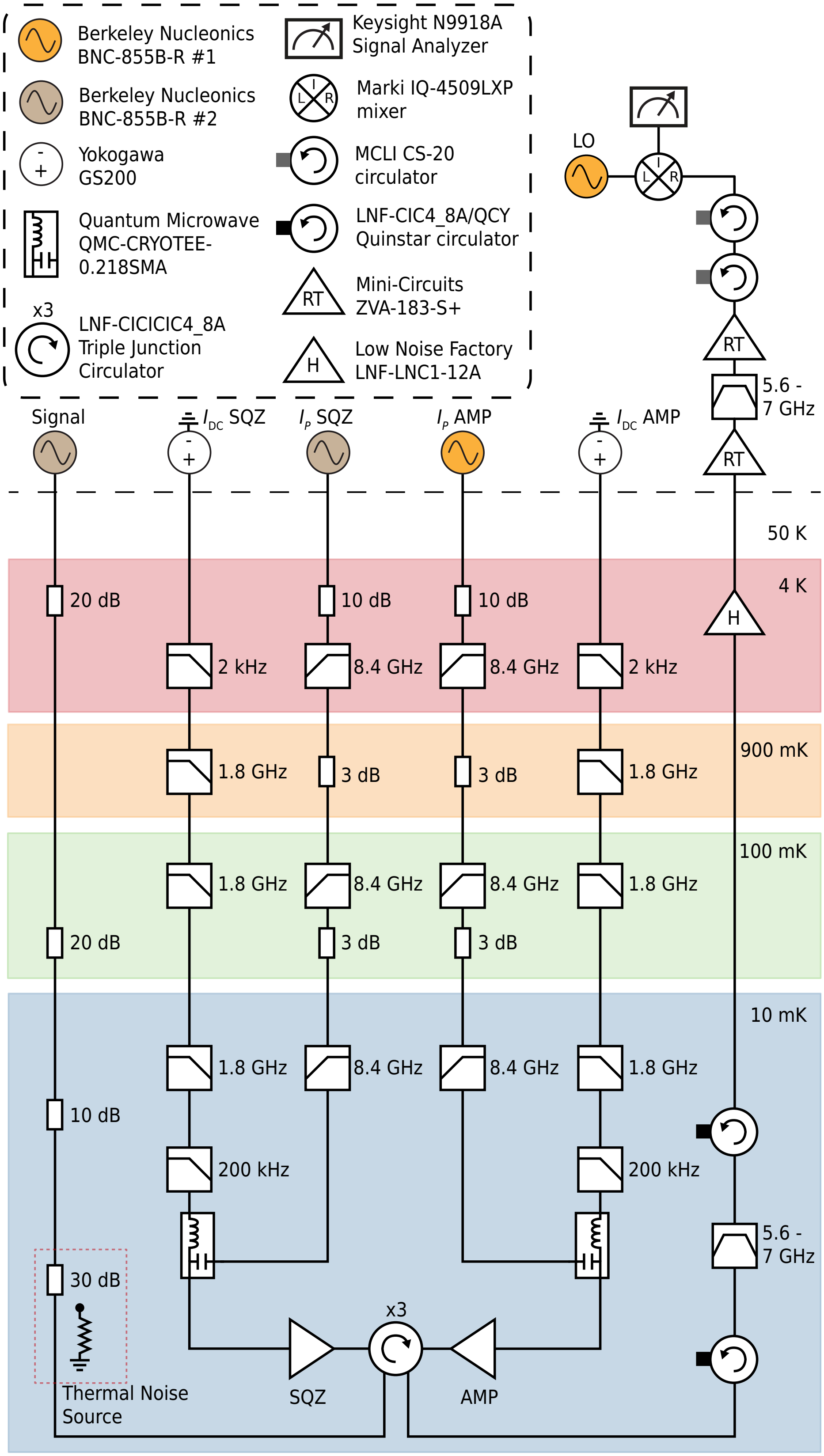

FIG. S1. A wiring diagram for the commercial dilution refrigerator used in experiments.

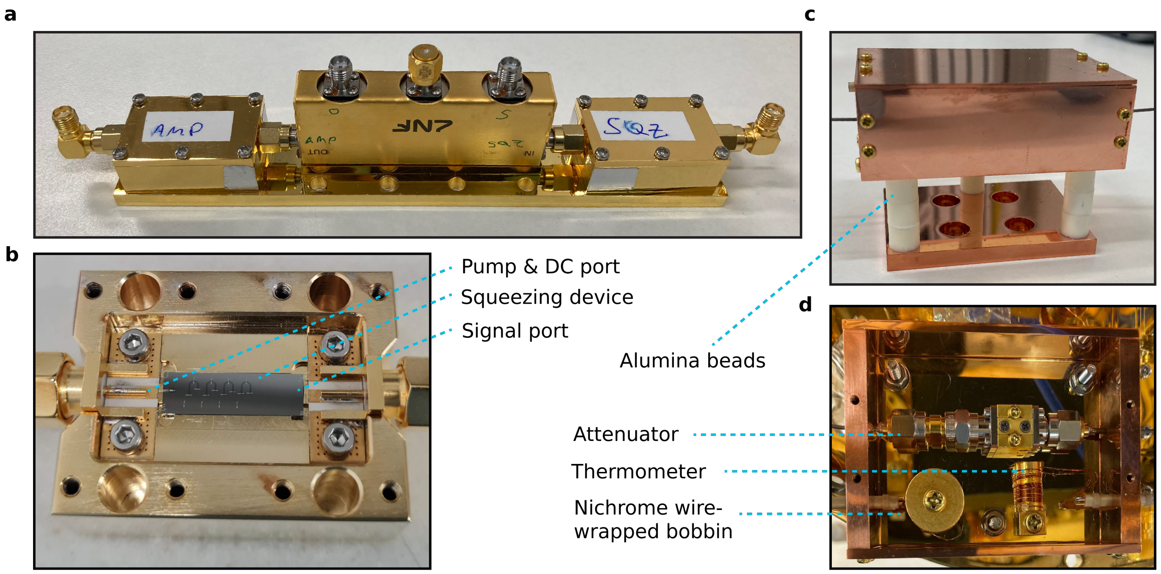

FIG. S2. Custom components used for squeezing measurements. **a**, The two-port KIPA design allows us to connect two KIPAs with only a single circulator between them. The device enclosures and circulator are mounted to a custom baseplate to ensure alignment of the SMA connectors. **b**, The bottom half of a KIPA enclosure with a squeezing device. **c**, The enclosure for the thermal noise source. The box is mounted on stainless steel screws with alumina beads to prevent heat transfer from the noise source to the mixing chamber plate. **d**, The inside of the thermal noise source, consisting of a 30 dB attenuator, a thermometer and a heater consisting of a gold-plated copper bobbin wrapped with nichrome wire.

1. SUPPLEMENTARY NOTE 1: EXPERIMENTAL SETUP

A. Dilution Refrigerator Wiring

A schematic depicting the lines of a commercial dilution refrigerator used in the experiments is shown in Fig. S1. SQZ and AMP are connected to a triple-junction circulator (Low Noise Factory LNF-CICICIC4-8A). The device enclosures and the circulator are mounted together on a custom-made gold-plated oxygen-free copper block which is thermally anchored to the mixing chamber (MXC) plate at 10 mK. This design allows the male SMA connectors of the device enclosures to connect directly to the female SMA connectors of the circulator to minimize insertion loss (Fig. S2a), and is the configuration we use for the main squeezing experiments. The circulator port prior to SQZ is used for injecting coherent signals produced via a room temperature ultra-low phase-noise microwave source (Berkeley Nucleonics BNC-845b) or thermal noise (see below). The final port of the circulator routes the signals to a HEMT at 4 K (Low Noise Factory LNF-LNC1-12A). Depending on the measurement, up to two additional room temperature amplifiers are used (Mini-Circuits ZVA-183-GX+).

A total of four lines are used to supply the pump and $I_{\rm DC}$ to each KIPA, with both pairs of lines nominally identical to one another. The lines for each control signal are extensively filtered across the various temperature stages to prevent thermal noise at ω_0 from reaching the KIPAs. The pump and $I_{\rm DC}$ signals are combined at the MXC with a bias tee (Quantum Microwave QMC-CRYOTEE-0.218SMA) and fed into one port of the device (see below). The pump tones are attenuated by 16 dB of fixed attenuation. Aside from the fixed attenuation, at the pump frequency of about 12 GHz the coaxial cables exhibit a non-negligible amount of loss. In Ref. [1], calibration measurements of the line loss in the same dilution refrigerator for a slightly different wiring configuration were performed. Based on this past calibration we estimate the line loss in this setup at the pump frequency to be around 30 dB. This is used to provide an estimate of the pump powers at the device input in Fig. 2 of the main text.

We use home-built low-pass filters for filtering the DC lines at 4 K and at the MXC. The low-pass filters at 4 K are made from a 40 Ω length of nichrome wire wrapped around a gold-plated copper bobbin with a total of 1.88 μF of shunt capacitance, thus realizing a series RC-filter with a 2 kHz cut-off. The low-pass filters used at the MXC employ the same total capacitance of 1.88 μF but with a low 0.4 Ω resistance copper wire (measured at room temperature) to avoid ohmic heating. These filters are enclosed in an aluminum box, with a copper base plate to enhance their thermal contact with the fridge. The inside of each box is lined with eccosorb (Laird Technologies EMI 21109145) to further attenuate signals at microwave frequencies. These homemade filters each attenuate $>$ 50 dB over the frequency range $\sim$ 200 kHz to 30 GHz.

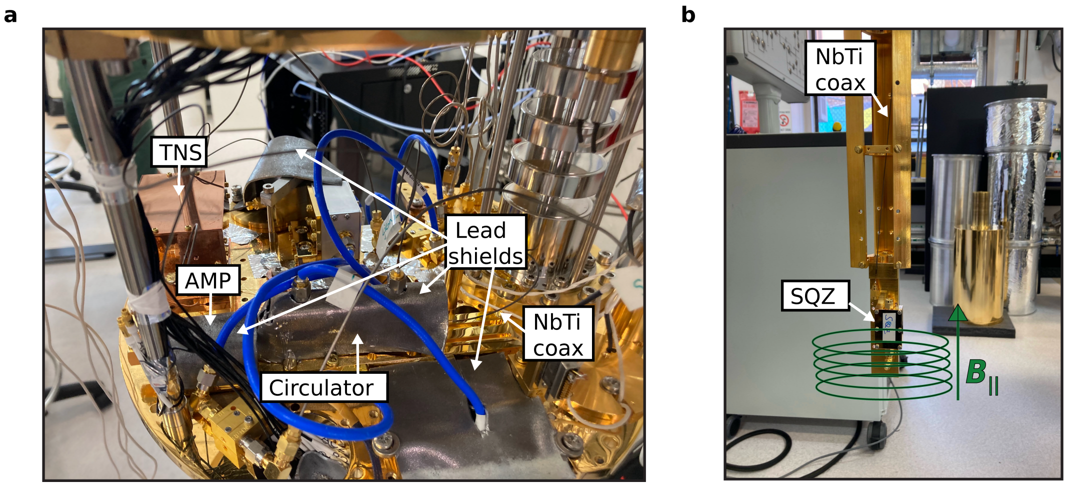

FIG. S3. Lead shielding for the experiments with a magnetic field. **a**, Lead shields on the mixing chamber plate partially protect the circulator and AMP from stray magnetic fields from the vector magnet. In the experiments with a magnetic field, SQZ is placed on the cold finger, connected to the circulator via a NbTi coaxial cable. **b**, SQZ is on the cold finger below the mixing chamber plate. Here, we schematically show the magnet coil (green rings) to clarify the direction of the magnetic field $B_{\|}$.

The wiring diagram shown in Fig. S1 illustrates the setup of the squeezing experiments presented in Fig. 3 in the main text. For the squeezing experiments performed in a magnetic field shown in Figs. 4a-c, we modified the setup slightly. As depicted in Fig. S3b, we installed a 6 T/1 T/1 T vector magnet and mounted SQZ in its center, connecting SQZ to the triple junction circulator with a 56 cm long superconducting NbTi coaxial cable. We emphasize that all other wiring and the placement of the AMP and the circulator remain unchanged. The circulator and the AMP were wrapped in 1 mm thick lead sheets (which are superconducting below a critical temperature of 7.2 K) to shield these components from the stray field expected to reach the mixing chamber plate (see Fig. S3a). Based on the specifications of the magnet, we estimate this stray field to be order 5 mT per 1 T applied in the vertical coil [2]. We note that the lead shielding was installed around the existing setup and therefore could not cover all relevant components entirely, potentially allowing some of the stray field through.

For the squeezing measurements as a function of temperature presented in Figs. 4d-g in the main text, we modified the setup such the SQZ and the triple junction circulator were mounted on the thermal noise source shown in Fig. S2c. A superconducting NbTi coaxial cable connected SQZ and the circulator to avoid creating a thermal link. The thermal noise source was thermally isolated from the mixing chamber plate, as described in Section 1 C, which ensured that SQZ and the circulator were thermally isolated from AMP. We again emphasize that that all other wiring and the placement of AMP remain unchanged.

B. Circulator Insertion Loss

The data in Fig. S4a depicts the typical insertion loss and isolation performance of single, double and triple junction ferrite circulators produced by the manufacturer Low Noise Factory. From this plot we estimate the insertion loss of the triple junction circulator used in our experiments to be -0.2 dB at cryogenic temperatures. The total insertion loss of the circulator can be decomposed into three primary components: insertion loss due to reflections from the connectors; loss from the internal solder joints; and loss from the garnet material that is used to provide the circulator with its nonreciprocal behavior. Here we break down the contribution of each of these elements.

From the port match plot shown in Fig. S4b, we estimate a return loss of 22 dB at $\omega/2\pi$=6.23 GHz at a temperature of 4 K. This corresponds to an insertion loss of approximately $10\log\left(1-10^{-22/10}\right)=-0.03$ dB per connector. The solder joint resistance and connector mated pair resistance contribute approximately 0.05 dB over the frequency range of 4-12 GHz per connector (private communication with Low Noise Factory). This leaves an estimated garnet dissipation loss of 0.04 dB. If the two KIPAs and triple-junction circulator were integrated in the same enclosure, the connector and solder joint insertion losses would be eliminated. Assuming garnet dissipation as the sole source of insertion loss and the amplifier-added noise was completely mitigated, squeezing would be limited to -20.4 dB.

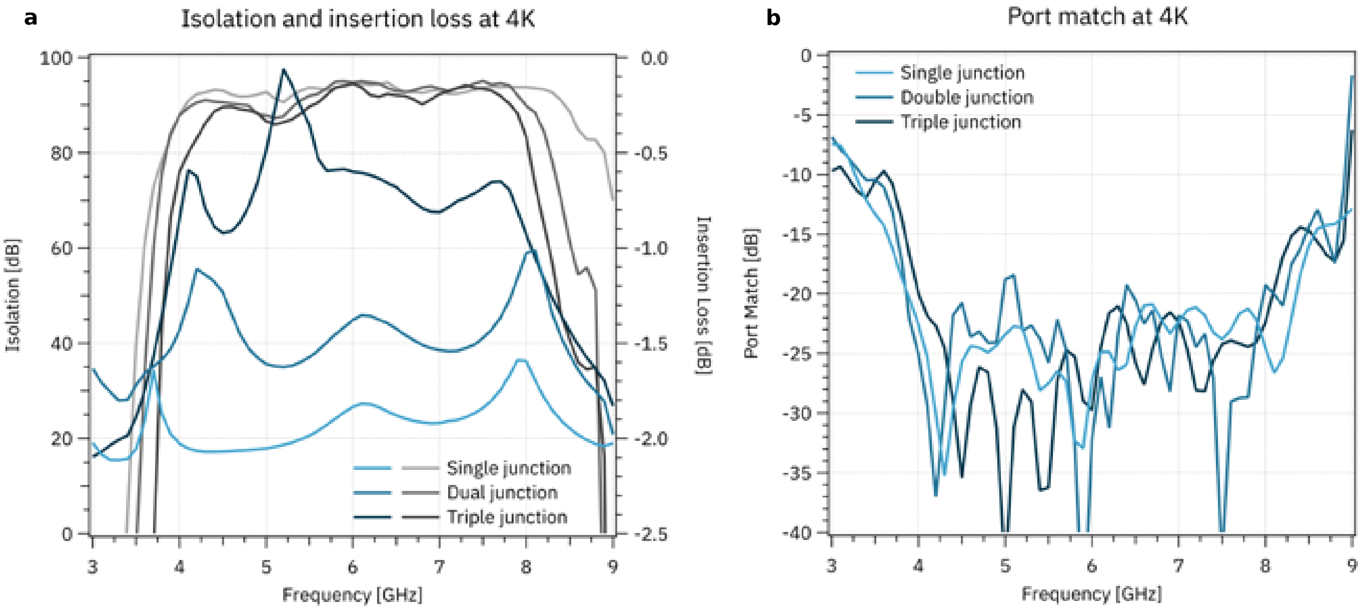

FIG. S4. **a**, Measurement of typical circulator isolation (blue lines) and insertion loss (grey lines) **b**, Typical port match of the Low Noise Factory 4-8 GHz circulators with single, dual and triple junction(s), plotted as a function of the signal frequency. Plots supplied by Low Noise Factory.

C. Thermal Noise Source

To calibrate the noise added by each KIPA we utilize a home-built variable temperature thermal noise source (TNS) (Fig. S2e). The design of the TNS is inspired by Simbierowicz, et al. [3] and consists of a 30 dB cryo-attenuator (Quantum Microwaves QMC-CRYOATT-30) that is heated by a nichrome wire wrapped around a copper bobbin. A low-temperature ruthenium oxide thermometer (Ice Oxford PKE0272) calibrated down to 30 mK is used to monitor the temperature of the TNS. The TNS is placed inside an oxygen-free copper box which is thermally isolated from the MXC via alumina beads wrapped around stainless steel screws. The thermal conductance of alumina rapidly diminishes below 3 K and thus serves to efficiently cool the noise source to this temperature. Below 3 K cooling predominantly occurs through the stainless steel mounting screws, which ensures that the noise source has sufficient thermal isolation from the MXC [3]. Superconducting NbTi cables are used to connect the heated attenuator to the circulator and input line to minimize loss and provide thermal isolation.

2. SUPPLEMENTARY NOTE 2: THEORY

In this section we present the input-output theory used to model the squeezing measurements in the squeezed-state receiver setup.

A. Input-Output Theory

1. *Single KIPA in Phase-Sensitive Mode*

For a single KIPA in phase-sensitive mode, the variance of the mode quadrature operators at its output (proportional to the noise power) can be directly related to the variance of the operators at its input via the input-output relations for the KIPA, which were derived in Appendix D of Ref. [4]. The quadrature operators for the input propagating mode are given by

$$I_{\mathrm{in}} = \frac{a_{\mathrm{in}} + a_{\mathrm{in}}^{\dagger}}{2}, \quad Q_{\mathrm{in}} = \frac{a_{\mathrm{in}} - a_{\mathrm{in}}^{\dagger}}{2i}, \tag{1}$$

where a_{in} and $a_{\text{in}}^\dagger$ are the bosonic annihilation and creation operators for the input mode, respectively. The input-output relations are then

$$\begin{pmatrix} I_{\text{out}} \\ Q_{\text{out}} \end{pmatrix} = \underbrace{\begin{pmatrix} A & B \\ C & D \end{pmatrix}}_{G} \begin{pmatrix} I_{\text{in}} \\ Q_{\text{in}} \end{pmatrix} + \sqrt{\frac{\gamma}{\kappa}} \underbrace{\begin{pmatrix} A+1 & B \\ C & D+1 \end{pmatrix}}_{G+1} \begin{pmatrix} I_b \\ Q_b \end{pmatrix}, \tag{2}$$

where γ is the internal loss rate and κ is the coupling rate to an external port. G is equal to

$$G(\phi) = \frac{\kappa}{\Delta^2 + \bar{\gamma}^2 - |\xi|^2} \begin{pmatrix} \bar{\gamma} - |\xi|\sin(\phi) & -|\xi|\cos(\phi) + \Delta \\ -|\xi|\cos(\phi) - \Delta & \bar{\gamma} + |\xi|\sin(\phi) \end{pmatrix} - 1, \tag{3}$$

where $\xi = -e^{-i2\phi_p}\omega_0 I_{\text{DC}} I_p/(4I_*^2)$ is the 3WM strength, $\Delta = \omega_0 - \omega_p/2$ is the frequency detuning, $\bar{\gamma} = (\kappa+\gamma)/2$ and I_b and Q_b are the quadrature operators for the bath mode that is coupled to the KIPA. They are similarly defined as

$$I_b = \frac{b_{\text{in}} + b_{\text{in}}^\dagger}{2}, \quad Q_b = \frac{b_{\text{in}} - b_{\text{in}}^\dagger}{2i}, \tag{4}$$

where b_{in} and $b_{\text{in}}^\dagger$ are the bosonic annihilation and creation operators for the bath mode, respectively,

Solving for the variance at the device output δI_{out}^2, we find

$$\delta I_{\text{out}}^2 = A^2(\delta I_{\text{in}}^2) + B^2(\delta Q_{\text{in}}^2) + \frac{\gamma}{\kappa}\left[(A+1)^2(\delta I_b^2) + B^2(\delta Q_b^2)\right]. \tag{5}$$

In Eq. 5 we have made use of the fact that the orthogonal quadratures of the input field are uncorrelated (i.e. $\langle I_{\text{in}}, Q_{\text{in}}\rangle = 0$) as are the input and bath fields (e.g. $\langle I_{\text{in}}, Q_b\rangle = 0$). Note that the variance of the I and Q quadratures are in general linked to one another, however, if $\Delta = \cos(\phi) = 0$ they become linearly independent. In this case,

$$\delta I_{\text{out}}^2 = G_{\text{K}}(\delta I_{\text{in}}^2) + (G_{\text{K}} - 1)n_{\text{K}}, \tag{6}$$

where $G_{\text{K}} = A^2$ is the KIPA power gain, with the subscript 'K' denoting KIPA. When $G_{\text{K}} > 1$, which occurs when $\phi = 3\pi/2$, the KIPA anti-squeezes the noise along I. When $0 \le G_{\text{K}} < 1$, which occurs when $\phi = \pi/2$, the KIPA squeezes the noise along I. n_{K} is the total number of noise photons added by the KIPA to the I quadrature and is equal to

$$n_{\text{K}} = \frac{\gamma}{\kappa}\frac{A+1}{A-1}\delta I_b^2 = \frac{\gamma}{\kappa}\frac{A+1}{A-1}\left(\frac{1}{4} + n_{\text{K,th}}\right), \tag{7}$$

where $n_{\text{K,th}}$ is the noise added by the KIPA per quadrature of b_{in} measured in excess of vacuum, in units of photons.

Equation 6 is intuitive in that by deactivating the KIPA, which is equivalent to setting $G_{\text{K}} = 1$, the input mode simply reflects off the KIPA without any noise being added. Note that taken in isolation, Eq. 7 seems to suggest that the noise added by the KIPA is negative when $A < 1$, i.e. when the KIPA is configured to squeeze. However, we see that this is accounted for in Eq. 6 because the term $G_{\text{K}} - 1 < 0$, such that the noise added to the I-quadrature is always greater than zero whenever $G_{\text{K}} \neq 1$.

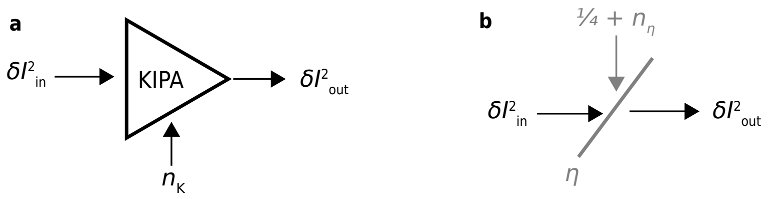

FIG. S5. **a**, Input-output model of a single KIPA, corresponding to Eq. 2. **b**, Input-output model of a beamsplitter, corresponding to Eq. 8, which is used to describe the finite insertion loss of components in the setup.

2. Input-Output Relation for the Loss Between Components

We can model the loss between two components in the setup as a beamsplitter with transmission efficiency η. The beamsplitter both attenuates the input mode and mixes in noise from the bath. The noise of the bath, stated in terms of noise photons, is given by $\delta I_\eta^2 = 1/4 + n_\eta$, where $n_\eta = [\exp(\hbar\omega_0/k_B T_\eta) - 1]^{-1}/2$ is the Bose-Einstein occupation (per quadrature) for a field with a frequency ω_0 and at a temperature T_η. The input-output relation for the beamsplitter is then

$$\begin{aligned}\delta I_{\text{out}}^2 &= \eta(\delta I_{\text{in}}^2) + (1-\eta)(\delta I_\eta^2) \\ &= \eta(\delta I_{\text{in}}^2) + (1-\eta)\left(\frac{1}{4} + n_\eta\right).\end{aligned} \tag{8}$$

3. Input-Output Relation for the Full Experimental Setup

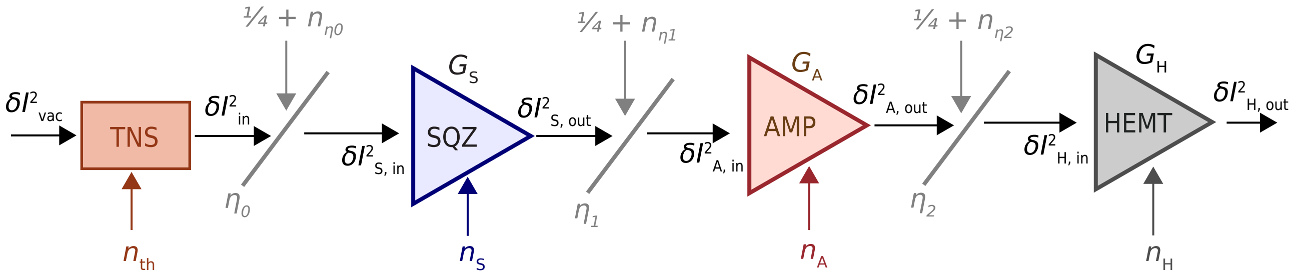

FIG. S6. Input-output diagram containing a Thermal Noise Source (TNS), two KIPAs (SQZ and AMP) and a HEMT amplifier. Each component is linked with a non-ideal transmission modelled as a beamsplitter with transmission efficiency η_i

The input-output relations for the full setup can be derived by linking the input-output relations for each discrete component and accounting for the finite insertion loss between them. An input-output diagram of the direct squeezing setup (Fig. 1 of the main text) is shown in Fig. S6. It is comprised of a thermal noise source (TNS), two KIPAs, and a High Electron Mobility Transistor (HEMT) amplifier, all connected in series via a circulator. The reader is referred to Fig. S6 for the definition of each term in the following input-output relations. We ignore the room temperature amplifiers in our setup (Fig. S1) since $G_H n_H$ far exceeds the noise added by these devices, or in other words, the noise in our measurements is dominated by sources within the dilution refrigerator.

We build a series of coupled linear equations, going from left to right in Fig. S6:

$$\delta I_{\text{in}}^2 = \delta I_{\text{vac}}^2 + n_{\text{th}} = \frac{1}{4} + n_{\text{th}} \tag{9}$$

$$\delta I_{\text{S,in}}^2 = \eta_0(\delta I_{\text{in}}^2) + (1-\eta_0)\left(\frac{1}{4} + n_{\eta_0}\right) \tag{10}$$

$$\delta I_{S\text{,out}}^2 = G_\text{S}(\delta I_{\text{S,in}}^2) + (G_\text{S} - 1)n_\text{S} \tag{11}$$

$$\delta I_{\text{A,in}}^2 = \eta_1(\delta I_{\text{S,out}}^2) + (1-\eta_1)\left(\frac{1}{4} + n_{\eta_1}\right) \tag{12}$$

$$\delta I_{\text{A,out}}^2 = G_\text{A}(\delta I_{A\text{,in}}^2) + (G_\text{A} - 1)n_\text{A} \tag{13}$$

$$\delta I_{\text{H,in}}^2 = \eta_2(\delta I_{\text{A,out}}^2) + (1-\eta_2)\left(\frac{1}{4} + n_{\eta_2}\right) \tag{14}$$

$$\delta I_{\text{H,out}}^2 = G_\text{H}(\delta I_{\text{H,in}}^2) + (G_\text{H} - 1)n_\text{H}. \tag{15}$$

In Eq. 9, $n_{\text{th}} = [\exp(\hbar\omega/k_B T_\text{H}) - 1]^{-1}/2$ is the thermal noise added by the TNS to the I quadrature. In Eq. 15, $n_\text{H} = 1/4 + n_{H,\text{th}}$ is the total noise added by the HEMT to the I-quadrature, where $n_{H,\text{th}}$ is the noise added by the HEMT in excess of vacuum.

Combining equations 9-15 we create a single input-output relation for the experimental setup

$$\begin{aligned}\delta I^2_{\mathrm{H,out}} &= G_\mathrm{H} G_\mathrm{A} G_\mathrm{S} \eta_0 \eta_1 \eta_2 \left(\frac{1}{4} + n_\mathrm{th}\right) \\ &+ G_\mathrm{H} G_\mathrm{A} G_\mathrm{S} (1-\eta_0) \eta_1 \eta_2 \left(\frac{1}{4} + n_{\eta 0}\right) \\ &+ G_\mathrm{H} G_\mathrm{A} (G_\mathrm{S}-1) \eta_1 \eta_2 n_\mathrm{S} \\ &+ G_\mathrm{H} G_\mathrm{A} (1-\eta_1) \eta_2 \left(\frac{1}{4} + n_{\eta 1}\right) \\ &+ G_\mathrm{H} (G_\mathrm{A}-1) \eta_2 n_\mathrm{A} \\ &+ N_\mathrm{sys},\end{aligned} \quad , \tag{16}$$

where for conciseness we group together the system noise N_sys (referred to the output of the HEMT amplifier), which is equal to

$$N_\mathrm{sys} = G_\mathrm{H}(1-\eta_2)\left(\frac{1}{4} + n_{\eta 2}\right) + (G_\mathrm{H}-1) n_\mathrm{H}. \tag{17}$$

The input-output relation in Eq. 16 is the theoretical framework for all of the experiments in the main text. In the next sections we apply it to determine what limits our measurements of direct squeezing, to define a procedure for calibrating the noise added by SQZ and AMP, and for explaining the thermal state squeezing experiments.

B. Limitations of the Direct Squeezing Measurements

As described in the main text, we make direct measurements of vacuum squeezing by comparing the power of the noise along I when both SQZ and AMP are activated and when only AMP is activated. Using Eq. 16, this measurement protocol is equal to

$$S = \frac{\delta I^2_{\mathrm{H,out}}\Big|_{G_s\neq 1, G_a\neq 1, n_\mathrm{th}=0}}{\delta I^2_{\mathrm{H,out}}\Big|_{G_s=1, G_a\neq 1, n_\mathrm{th}=0}} := \frac{\delta I^2_\mathrm{S,A}}{\delta I^2_\mathrm{A}}. \tag{18}$$

We assume that the bath temperatures for the losses η_0 and η_1 are both of the order of $T_\mathrm{MC} \approx 10$ mK, which ensures that $n_{\eta_0} = n_{\eta_1} \approx 0$. The numerator of Eq. 18 then becomes

$$\delta I^2_\mathrm{S,A} = G_\mathrm{H} G_\mathrm{A} \eta_2 \left[(G_\mathrm{S}-1)\eta_1 \left(\frac{1}{4} + n_\mathrm{S}\right) + \frac{1}{4} + n_\mathrm{A}\left(1 - \frac{1}{G_\mathrm{A}}\right)\right] + N_\mathrm{sys}, \tag{19}$$

and the denominator becomes

$$\delta I^2_\mathrm{A} = G_\mathrm{H} G_\mathrm{A} \eta_2 \left[\frac{1}{4} + n_\mathrm{A}\left(1 - \frac{1}{G_\mathrm{A}}\right)\right] + N_\mathrm{sys}. \tag{20}$$

Combining these expressions we find

$$S = 1 - \frac{\eta_1 (1-G_\mathrm{S})(1/4 - |n_\mathrm{S}|)}{1/4 + (1-1/G_\mathrm{A}) n_\mathrm{A} + N_\mathrm{sys}/(G_\mathrm{H} G_\mathrm{A} \eta_2)}, \tag{21}$$

where we have taken the absolute value of n_S in the second line to clarify that when $G_\mathrm{S} < 1$, $n_\mathrm{S} < 0$ (see Sec. 2 A 1). Provided $|n_\mathrm{S}| < 1/4$, we see that Eq. 21 predicts that activating SQZ should reduce the noise measured at the output of the system.

In the limit of high AMP gain ($N_\mathrm{sys}/G_\mathrm{A} G_\mathrm{H} \eta_2 \ll 1/4$ and $G_\mathrm{A} \gg 1$), i.e. when the system noise referred to the input of AMP is sufficiently smaller than the vacuum fluctuations, this expression reduces to Eq. 1 in the main text. Equation 21 exposes the two main experimental limitations in the direct measurement of squeezing: the insertion loss between SQZ and AMP, η_1, and the noise added by the amplifiers, n_S and n_A.

C. Amplifier Noise Calibration

We calibrate the noise added by both SQZ and AMP by using the TNS to vary the thermal occupation of the input state n_{th}. Crucially, the KIPAs remain stable at ~ 10 mK. The noise added by the KIPAs n_{S} and n_{A} can then be found through the relation between the power of the noise measured at the output of the system and n_{th}.

To find n_{S} and n_{A} we measure $\delta I^2_{\mathrm{H,out}}$ as a function of n_{th} in three different scenarios: SQZ activated ($G_{\mathrm{A}} = 1$), AMP activated ($G_{\mathrm{S}} = 1$), and both SQZ and AMP deactivated ($G_{\mathrm{A}}, G_{\mathrm{S}} = 1$). For each scenario, using Eq. 16 we obtain a linear relation between $\delta I^2_{\mathrm{H,out}}$ and n_{th} and find slopes that are given by

$$s_{\mathrm{S}} := \left.\frac{d\delta I^2_{\mathrm{H,out}}}{dn_{\mathrm{th}}}\right|_{G_{\mathrm{A}}=1} = G_{\mathrm{H}} G_{\mathrm{S}} \eta_0 \eta_1 \eta_2, \tag{22}$$

$$s_{\mathrm{A}} := \left.\frac{d\delta I^2_{\mathrm{H,out}}}{dn_{\mathrm{th}}}\right|_{G_{\mathrm{S}}=1} = G_{\mathrm{H}} G_{\mathrm{A}} \eta_0 \eta_1 \eta_2, \tag{23}$$

$$s_{\mathrm{off}} := \left.\frac{d\delta I^2_{\mathrm{H,out}}}{dn_{\mathrm{th}}}\right|_{G_{\mathrm{S}}=G_{\mathrm{A}}=1} = G_{\mathrm{H}} \eta_0 \eta_1 \eta_2, \tag{24}$$

and with y-intercepts

$$\begin{aligned} y_{\mathrm{S}} := \delta I^2_{\mathrm{H,out}}\big|_{G_{\mathrm{A}}=1, n_{\mathrm{th}}=0} =& \frac{G_{\mathrm{H}} G_{\mathrm{S}} \eta_0 \eta_1 \eta_2}{4} + G_{\mathrm{H}} G_{\mathrm{S}} (1-\eta_0) \eta_1 \eta_2 \left(\frac{1}{4} + n_{\eta 0}\right) \\ & + G_{\mathrm{H}} (G_{\mathrm{S}} - 1) \eta_1 \eta_2 n_{\mathrm{S}} + G_{\mathrm{H}} (1-\eta_1) \eta_2 \left(\frac{1}{4} + n_{\eta 1}\right) + N_{\mathrm{sys}} \end{aligned}, \tag{25}$$

$$\begin{aligned} y_{\mathrm{A}} := \delta I^2_{\mathrm{H,out}}\big|_{G_{\mathrm{S}}=1, n_{\mathrm{th}}=0} =& \frac{G_{\mathrm{H}} G_{\mathrm{A}} \eta_0 \eta_1 \eta_2}{4} + G_{\mathrm{H}} G_{\mathrm{A}} (1-\eta_0) \eta_1 \eta_2 \left(\frac{1}{4} + n_{\eta 0}\right) \\ & + G_{\mathrm{H}} G_{\mathrm{A}} (1-\eta_1) \eta_2 \left(\frac{1}{4} + n_{\eta 1}\right) + G_{\mathrm{H}} (G_{\mathrm{A}} - 1) \eta_2 n_{\mathrm{A}} + N_{\mathrm{sys}} \end{aligned}, \tag{26}$$

$$\begin{aligned} y_{\mathrm{off}} := \delta I^2_{\mathrm{H,out}}\big|_{G_{\mathrm{S}}=G_{\mathrm{A}}=1, n_{\mathrm{th}}=0} =& \frac{G_{\mathrm{H}} \eta_0 \eta_1 \eta_2}{4} + G_{\mathrm{H}} (1-\eta_0) \eta_1 \eta_2 \left(\frac{1}{4} + n_{\eta 0}\right) \\ & + G_{\mathrm{H}} (1-\eta_1) \eta_2 \left(\frac{1}{4} + n_{\eta 1}\right) + N_{\mathrm{sys}} \end{aligned}. \tag{27}$$

We now define a new parameter $\delta \tilde{I}^2_{\mathrm{k}} := \delta I^2_{\mathrm{k}} - \delta I^2_{\mathrm{off}}$, where $k \in \{S, A\}$, i.e. the difference between the noise measured with SQZ or AMP on with the noise measured when both SQZ and AMP are deactivated. For $\delta \tilde{I}^2_{\mathrm{S}}$, we obtain a y-intercept of

$$\begin{aligned} \tilde{y}_{\mathrm{S}} = y_{\mathrm{S}} - y_{\mathrm{off}} =& \frac{G_{\mathrm{H}} (G_{\mathrm{S}} - 1) \eta_0 \eta_1 \eta_2}{4} + G_{\mathrm{H}} (G_{\mathrm{S}} - 1)(1-\eta_0) \eta_1 \eta_2 \left(\frac{1}{4} + n_{\eta 0}\right) \\ & + G_{\mathrm{H}} (G_{\mathrm{S}} - 1) \eta_1 \eta_2 n_{\mathrm{S}} \\ =& \, G_{\mathrm{H}} (G_{\mathrm{S}} - 1) \eta_1 \eta_2 \left[\frac{1}{4} + (1-\eta_0) n_{\eta 0} + n_{\mathrm{S}}\right] \end{aligned} \tag{28}$$

and a slope of

$$\tilde{s}_{\mathrm{S}} = s_{\mathrm{S}} - s_{\mathrm{off}} = G_{\mathrm{H}} (G_{\mathrm{S}} - 1) \eta_0 \eta_1 \eta_2. \tag{29}$$

Similarly, for the case where AMP is activated, we find a y-intercept of

$$\begin{aligned}\tilde{y}_\mathrm{A} = y_\mathrm{A} - y_\mathrm{off} =& \frac{G_\mathrm{H}(G_\mathrm{A}-1)\eta_0\eta_1\eta_2}{4} + G_\mathrm{H}(G_\mathrm{A}-1)(1-\eta_0)\eta_1\eta_2\left(\frac{1}{4}+n_{\eta 0}\right)\\ &+ G_\mathrm{H}(G_\mathrm{A}-1)(1-\eta_1)\eta_2\left(\frac{1}{4}+n_{\eta 1}\right) + G_\mathrm{H}(G_\mathrm{A}-1)\eta_2 n_\mathrm{A}\\ &= G_\mathrm{H}(G_\mathrm{A}-1)\eta_2\left[\frac{1}{4}+(1-\eta_0)n_{\eta 0}+(1-\eta_1)n_{\eta 1}+n_\mathrm{A}\right]\end{aligned} \tag{30}$$

and a slope of

$$\tilde{s}_\mathrm{A} = s_\mathrm{A} - s_\mathrm{off} = G_\mathrm{H}(G_\mathrm{A}-1)\eta_0\eta_1\eta_2. \tag{31}$$

Dividing the y-intercept and the slope allows us to extract the noise added by SQZ and AMP, n_S and n_A, respectively. For 'SQZ on', we obtain

$$\frac{\tilde{y}_\mathrm{S}}{\tilde{s}_\mathrm{S}} = \frac{1}{\eta_0}\left[\frac{1}{4}+(1-\eta_0)n_{\eta 0}+n_\mathrm{S}\right], \tag{32}$$

and for 'AMP on' we obtain

$$\frac{\tilde{y}_\mathrm{A}}{\tilde{s}_\mathrm{A}} = \frac{1}{\eta_0\eta_1}\left[\frac{1}{4}+(1-\eta_0)n_{\eta 0}+(1-\eta_1)n_{\eta 1}+n_\mathrm{A}\right]. \tag{33}$$

Using this model we determine the noise added by SQZ and AMP in Section 4 J.

D. Squeezing of Thermal States

For the experiments at elevated temperatures, described in Figs. 4d-g of the main text, the setup is modified such that SQZ and the circulator are thermally connected to the TNS. The temperatures for the beamsplitters η_0 and η_1 are assumed to be the same as the temperature of the input signal, i.e., $n_{\eta_0} = n_{\eta_1} = n_\mathrm{th}$. In this case, Eq. 16 simplifies to

$$\delta I^2_\mathrm{H,out} = G_\mathrm{H}G_\mathrm{A}\eta_2\left[(G_\mathrm{S}-1)\eta_1(\frac{1}{4}+n_\mathrm{th}+n_\mathrm{S}) + 1/4 + n_\mathrm{th} + \left(1-\frac{1}{G_\mathrm{A}}\right)n_\mathrm{A}\right] + N_\mathrm{sys}. \tag{34}$$

Equation 21 can similarly be re-written as

$$S = \frac{\delta I^2_\mathrm{S,A}(n_\mathrm{th})}{\delta I^2_\mathrm{A}(n_\mathrm{th})} = 1-(1-G_\mathrm{S})\eta_1\frac{1/4+n_\mathrm{th}+n_\mathrm{S}}{1/4+n_\mathrm{th}+(1-1/G_\mathrm{A})n_\mathrm{A}+N_\mathrm{sys}/G_\mathrm{H}G_\mathrm{A}\eta_2}. \tag{35}$$

In the high AMP gain limit ($N_\mathrm{sys}/G_\mathrm{A}G_\mathrm{H}\eta_2 \ll 1/4$ and $G_\mathrm{A} \gg 1$), this expression reduces to

$$S = \frac{\delta I^2_\mathrm{S,A}(n_\mathrm{th})}{\delta I^2_\mathrm{A}(n_\mathrm{th})} = 1-(1-G_\mathrm{S})\eta_1\frac{1/4+n_\mathrm{th}+n_\mathrm{S}}{1/4+n_\mathrm{th}+n_\mathrm{A}} = 1-(1-G_\mathrm{S})\eta_1\frac{1/4+n_\mathrm{th}-|n_\mathrm{S}|}{1/4+n_\mathrm{th}+n_\mathrm{A}}. \tag{36}$$

We fit the data in Supplementary Fig. S19 in the main text to this expression to extract η_1 and the noise added by SQZ when operated in squeezing mode n_S^sq.

3. SUPPLEMENTARY NOTE 3: DEVICE FABRICATION, DESIGN, AND PACKAGING

A. Fabrication

The two KIPAs in our experiment are nominally identical in their design and fabrication. They are fabricated from a 15 nm thin film of NbTiN deposited on high-resistivity silicon (> 10 kΩ·cm). They are patterned with a single step of electron beam lithography and subsequently etched with a CF_4:Ar plasma. The kinetic inductance of the film was calibrated to be $L_{k,0} = 17.8$ pH/□ by fabricating a capacitively coupled quarter-wavelength ($\lambda/4$) coplanar waveguide (CPW) resonator, and matching its measured resonant frequency with electromagnetic simulations of the device using the software package Sonnet.

B. Design

The devices are designed to have a half-wavelength ($\lambda/2$) resonator connected to two ports. One of the ports connects directly to the resonator via a band-stop stepped impedance filter (BS-SIF, left side in Fig. S7a), while the other is inductively coupled to the resonator (Fig. S7b) via a low-pass stepped impedance filter (LP-SIF, right side in Fig. S7a). A simplified circuit model of the device is presented in Fig. S7c. We employ a two-port design because it allows the pump tone, DC current I_{DC} and resonant tones (including squeezed states) to be independently routed off-chip. This is essential for squeezing experiments because it allows for the connection of SQZ and AMP in series with only a single intervening circulator. This ensures a minimum amount of insertion loss, which would otherwise degrade the squeezed states as they travel between SQZ and AMP.

The resonator contains a dense interdigitated capacitance (IDC) to ground (Fig. S7b), which lowers its impedance and reduces its physical size, relative to a CPW design. Lowering the impedance of the resonator improves the performance of the amplifier by increasing the pump current for a given power. This results in a greater modulation of the kinetic inductance and therefore higher gain. The resonator can be viewed as a lumped-element (or "artificial") transmission line with repeating unit cells. Here each unit cell consists of the capacitance C to ground of one set of fingers and the inductance L from the thin wire connecting one set of fingers to the next. For frequencies far below the associated low-pass cutoff frequency $f_c = 1/(\pi\sqrt{LC})$ of the unit cell, the resonator behaves like a conventional transmission line [5, 6]. From the measured resonance frequency $\omega_0/2\pi \approx 6.2$ GHz of the resonator, the impedance listed in Table S1 $Z_0 = 101.7\ \Omega$ and the total number of unit cells $N \approx 19$, we extract $L = Z_0/(N\omega_0) = 137.4$ pH and $C = 1/(N\omega_0 Z_0) = 13.3$ fF. From this we estimate $f_c = 235.6$ GHz, orders of magnitude above $\omega_0/2\pi$.

The BS-SIF is constructed from a total of nine $\lambda/4$ CPW segments, with alternating low-impedance (Z_l, five segments) and high-impedance (Z_h, four segments) [7]. The resonator is directly connected to a Z_l segment, which in combination with the inductive shunt on its opposite end, results in electrical boundary conditions that give rise to a $\lambda/2$ resonant mode. The purpose of the BS-SIF is to pass both a DC current I_{DC} and pump current I_{p} to the resonator, while strongly attenuating at the resonance frequency $\omega_0/2\pi$.

The LP-SIF is constructed from a total of five segments of CPW with alternating Z_h (three segments) and Z_l (two segments). The LP-SIF is introduced to filter out the pump tone at $\omega_p \approx 2\omega_0$ in order to limit crosstalk between SQZ and AMP (see Fig. S7e). It is implemented on chip using the NbTiN film to minimize insertion loss. Further, the LP-SIF is designed to be 50 Ω-matched at $\omega_0/2\pi$ to minimize reflections from the signal port.

The BS-SIF and LP-SIF are designed using ABCD matrices [5] and electromagnetic simulations (Sonnet). For each segment, we simulate CPW waveguides with centre conductor width W and a gap to ground width G, from which we extract the effective dielectric constant ϵ_r and characteristic impedance Z (see Table S1). This allows us to relate the electrical and physical lengths of each segment, so that a numerical model of each filter can be constructed using ABCD matrices. The numerical calculation of the BS-SIF transmission response is shown in Fig. S7d. It demonstrates a strong attenuation for frequencies near ω_0, and pass bands at both DC and the pump frequency. The numerical calculation of the LP-SIF filter response is shown in Fig. S7e. It contains the desired features of minimal attenuation around $\omega_0/2\pi \approx 6$ GHz followed by a sharp roll-off, which yields ∼20 dB attenuation at $\omega_p/2\pi \approx 12$ GHz.

The resonator is coupled to the LP-SIF via a shunt inductance, corresponding to two parallel fingers that connect to the ground plane of the device (purple dashed box in Fig. S7b). The inductive coupling provides a path to ground for the DC current I_{DC}, while simultaneously allowing control of the coupling quality factor over several orders of magnitude [8]. The dimensions of the fingers (12 μm wide and 248 μm long) were designed based on electromagnetic simulations (Sonnet) of the device with a targeted coupling quality factor $Q_c = 200$. Combining simulations with measurements of Q_c across several device iterations, the final device design yielded a Q_c in close agreement with the targeted value, see Fig. S8. This ensures that the KIPA is strongly over-coupled ($Q_i \gg Q_c$) when measured in reflection via this port.

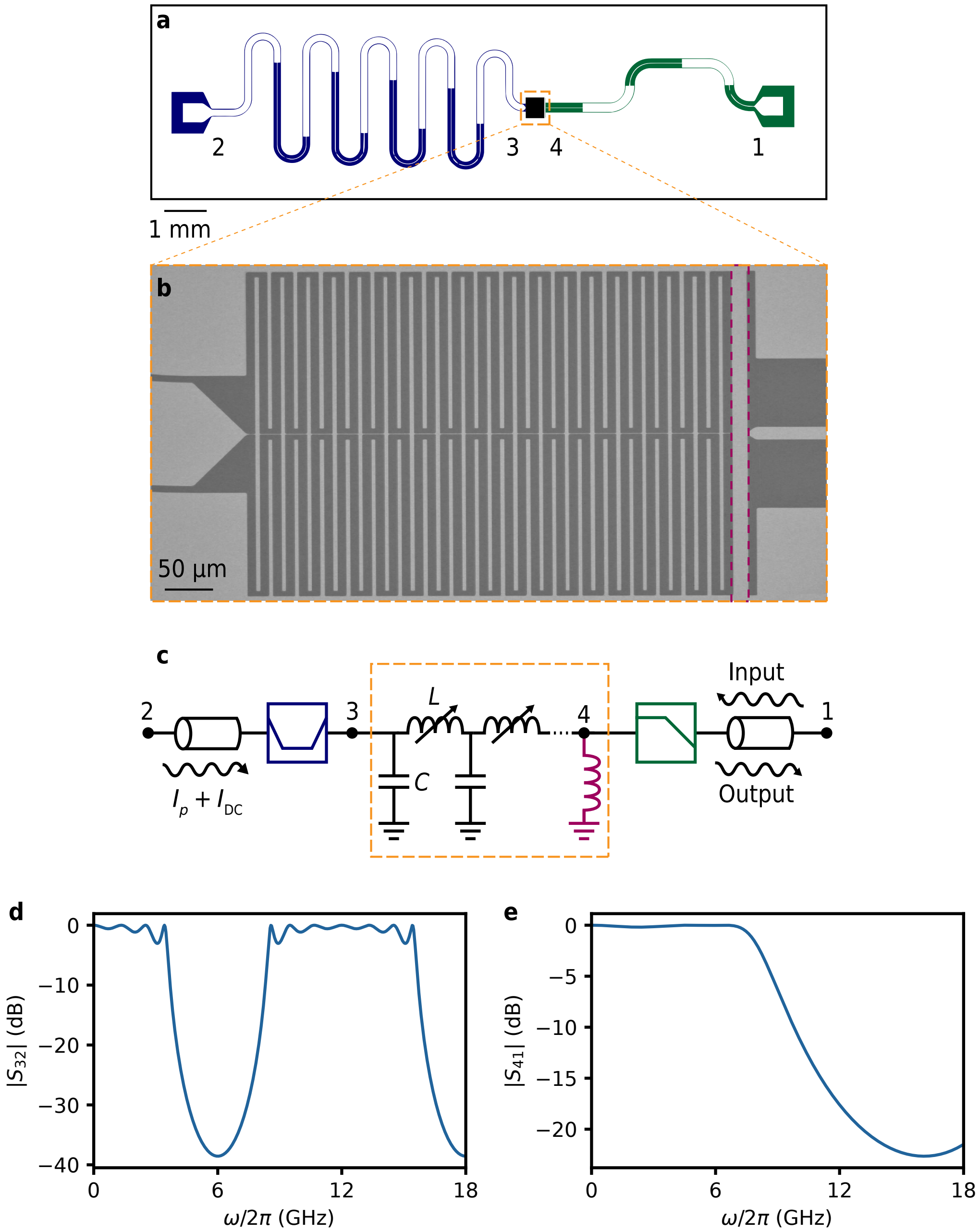

FIG. S7. **Design of the KIPAs.** **a**, The device layout. The right port (1) connects to a low-pass stepped-impedance filter (LP-SIF, green). The left port (2) connects to a band-stop stepped impedance filter (BS-SIF, blue). Both the LP-SIF and BP-SIF are constructed from alternating high Z_h- and low Z_l-impedance CPW segments. The half-wavelength resonator (black) is constructed from a CPW with a large interdigitated capacitance (IDC) to ground and is positioned in the center of the device. **b**, An optical image of the resonator. The shunt inductance controlling the coupling quality factor Q_c is highlighted (purple dashed box). **c**, Simplified circuit diagram of the device highlighting the role of the three core elements of the devices, the BS-SIF (blue) acts as a bandpass filter to attenuate at the resonance frequency ω_0, while permitting DC and pump tones to reach the device. The LP-SIF acts as a low-pass filter to reduce pump leakage at the signal port 1, while permitting signal at ω_0. The resonator (black) can be viewed as an artificial transmission line, with unit cell inductance L (which is nonlinear, as indicated with the arrow) and unit cell capacitance C. The orange highlighted region corresponds to the regions indicated in **a** and **b**, where the resonator shares an inductance to ground (purple) with the LP-SIF to couple the signal out of the resonator. **d**, The scattering parameter $|S_{32}|$ of the BS-SIF, numerically calculated using ABCD matrices. **e**, The scattering parameter $|S_{41}|$ of the LP-SIF, numerically calculated using ABCD matrices.

Parameter	Z_h (BS-SIF)	Z_l (BS-SIF)	Z_h (LP-SIF)	Z_l (LP-SIF)	IDC Resonator
Z (Ω)	131.1	38.7	137.0	34.3	101.7
ϵ_r	14.7	12.0	14.1	11.8	3237.6
W (μm)	20	160	20	214	1
G (μm)	80	10	105	8	10
length (μm)	3264	3607	680, 1214, 680	1692, 1692	452

TABLE S1. Design and simulated parameters of the various KIPA components. The resonator has an interdigitated capacitance (IDC) to ground consisting of 19 fingers with length 241 μm, width 4 μm and gap 8 μm.

C. Packaging

The KIPAs are mounted in separate device enclosures which are machined from gold-plated oxygen-free copper (Figs. S2b,c). The devices sit in a trench on top of a 500 μm thick piece of sapphire. The sapphire acts as a low-loss dielectric spacer to ensure that the resonant mode is sufficiently separated from metallic enclosure, whilst still maintaining good thermalization. The lid of the enclosure is designed so that the cavity sits within a 3D-waveguide whose cut-off frequency is 8 GHz, which is above the design of resonator ($\omega_0/2\pi = 6$ GHz). To ensure there are no gaps which might compromise the integrity of the waveguide mode, 1.5 cm long grooves are made in the enclosure 300 μm away from the long edge on either side of the device. In the grooves we place a 200 μm diameter indium wire which seals the waveguide when compressed by the enclosure lid. The device is grounded to the enclosure with wire bonds from the ground plane to the enclosure. Additional wire bonds are added to equalize the ground planes at each impedance step in the BS-SIF and LP-SIF to prevent the excitation of slotline modes.

The devices are bonded to PCBs made from gold-plated copper on Rogers RO3035 laminate. The dielectric constant of this material is known to have excellent thermal stability, which is important to ensure it remains impedance matched when cooled to low temperatures. The PCBs were designed to have the minimum length necessary to facilitate the PCB-mounted SMA connector and wire bonding.

4. SUPPLEMENTARY NOTE 4: CHARACTERIZATION OF THE KIPAS

In this section, we compare the operation and performance of the two KIPAs used in our experiments.

A. Reflection Measurement

To extract the resonance frequency $\omega_0/2\pi$, internal quality factor Q_i and the coupling quality factor Q_c of the each KIPA, we perform a reflection measurement (measured from port 1 in Figs. S7a,c) of the magnitude $|S_{11}|$ and phase response $\angle S_{11}$ of the resonator. We then extract ω_0, Q_i and Q_c from a combined fit [9]. An example of this using SQZ is shown in Figs. S8a,b.

B. Frequency Tunability

Both KIPAs exhibit closely-matched resonance frequencies at zero current, with SQZ having $\omega_0/2\pi(I_{\rm DC} = 0) =$ 6.341 GHz and AMP having $\omega_0/2\pi(I_{\rm DC} = 0) = 6.378$ GHz. In Fig. 2a of the main text we demonstrate that $\omega_0/2\pi$ of SQZ and AMP can be tuned by up to 215 MHz and 290 MHz respectively, using a DC current $I_{\rm DC}$. The $I_{\rm DC}$ enhances the kinetic inductance of the device according to the relation [10]

$$L_k(I_{\rm DC}) = L_{k,0}\left(1 + \frac{I_{\rm DC}^2}{I_*^2} + \mathcal{O}(I_*^4)\right), \tag{37}$$

where $L_{k,0}$ is the kinetic inductance at zero current and I_* is a constant that dictates the strength of the non-linearity. The frequency tunability of these devices is ultimately limited by their critical current I_c, which was found to be 1.22 mA for SQZ and 1.35 mA for AMP. I_* can be determined by fitting $\omega_0(I_{\rm DC})$ with the expression [4]

$$\omega_0(I_{\rm DC}) = \omega_0(0)\left(1 - \frac{I_{\rm DC}^2}{2I_*^2}\right), \tag{38}$$

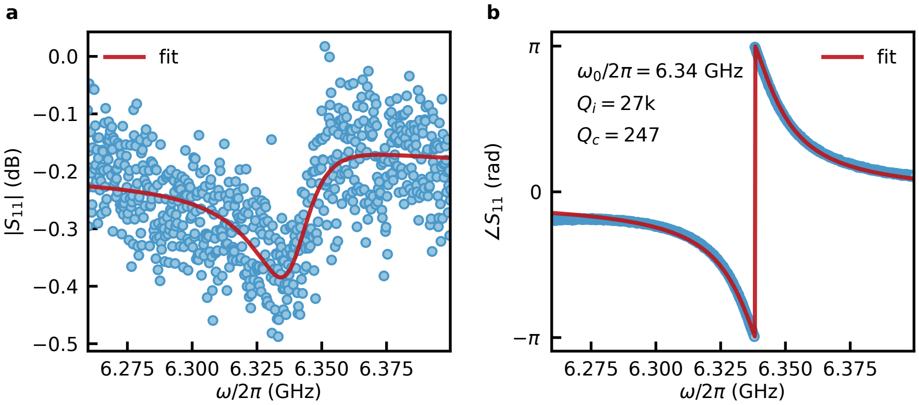

FIG. S8. **Reflection measurement of SQZ. a,b**, Magnitude $|S_{11}|$ (**a**) and phase $\angle S_{11}$ (**b**) response of SQZ measured as a function of probe frequency ω in reflection (S11) with a vector network analyzer. The solid red lines correspond to a fit of the data in the complex plane, from which we obtain ω_0, Q_i, and Q_c. The measurement is performed at $I_{\rm DC} = 0$ and $B_{\parallel} = 0$.

and was found to be $I_* = 5.1$ mA for both SQZ and AMP.

C. Gain

We compare the phase-insensitive gain of the two KIPAs by tuning them to the same resonance frequency $\omega_0/2\pi =$ 6.23 GHz with a DC current, pumping them with a microwave tone with frequency $\omega_p = 2\omega_0$, and measuring the scattering parameter S_{11} using a vector network analyzer, one device at a time. Figures S9a,b show the corresponding gain curves for SQZ and AMP measured as a function of pump power P_p. A baseline was subtracted from the S_{11} measurements, which was obtained by shifting the KIPAs' resonant frequencies outside of the measurement range with a DC current. The two devices perform in a near-identical manner, with SQZ and AMP achieving a peak phase-insensitive gain of 41.5 dB and 42.0 dB, respectively.

To compare the phase-sensitive gain of the KIPAs, we amplify coherent states with frequency ω_0 by pumping the devices with tones of frequency $\omega_p = 2\omega_0$ and variable phase (Figs. S9c,d). SQZ and AMP achieve nearly identical maximal phase-sensitive amplification (deamplification) of 50.5 dB and 50.9 dB (-13.6 dB and -12.2 dB), respectively. We attribute the large asymmetry in amplification and deamplification to be predominantly the result of the course step size in the phase used in this measurement.

D. Gain-Bandwidth Product

From the gain curves in Figures S9a,b we determine the gain-bandwidth product (GBP) for SQZ and AMP to be 17 MHz and 15 MHz, respectively. We note that these GBPs are a factor ~ 3 smaller than achieved in a previous device [4], which provides confidence that this can be improved on in the future.

In a resonant degenerate parametric amplifier (DPA), it can be shown that the GBP is set by the resonator linewidth $\kappa_L = \omega_0/Q_L$ (where $Q_L = (1/Q_c + 1/Q_i)^{-1}$ is the loaded quality factor) [11]. Gain in a DPA is produced as the pump strength, equal to $|\xi| = \omega_0 I_{DC} I_p/(4I_*^2)$ for our KIPAs [4], is increased towards the instability point $|\xi| = \kappa_L/2$ [4, 12]. This sets an upper bound for the GBP of $\kappa_L = 2|\xi|$. In the present devices, we opted for smaller resonator linewidths κ_L (or larger quality factors) compared to our previous work in order to reduce the pump current I_p (and therefore pump power) needed to operate the devices. This minimized any pump-induced heating that could have restricted the amount of vacuum squeezing.

To increase the GBP we must raise κ_L, which implies higher pump currents. We believe that we are already operating near the upper limit of the pump powers that can be applied in such delicate experiments as squeezing, thus strategies for increasing I_p without raising the pump power must be adopted. We can increase I_p for a given pump power by lowering the resonator impedance (e.g. by increasing the interdigitated capacitance) [4]. In addition, we can reduce the I_p required by lowering the resonator I_*, which could readily be achieved by decreasing the resonator wire width or the film thickness [13]. Since I_* is proportional to the critical current (I_c) of the device [10], where in the present study $I_* \approx 5I_c$, then the upper limit for the pump strength is $|\xi| \approx \omega_0 \times 1/25 \times 1/4 = 2\pi \times 62$ MHz.

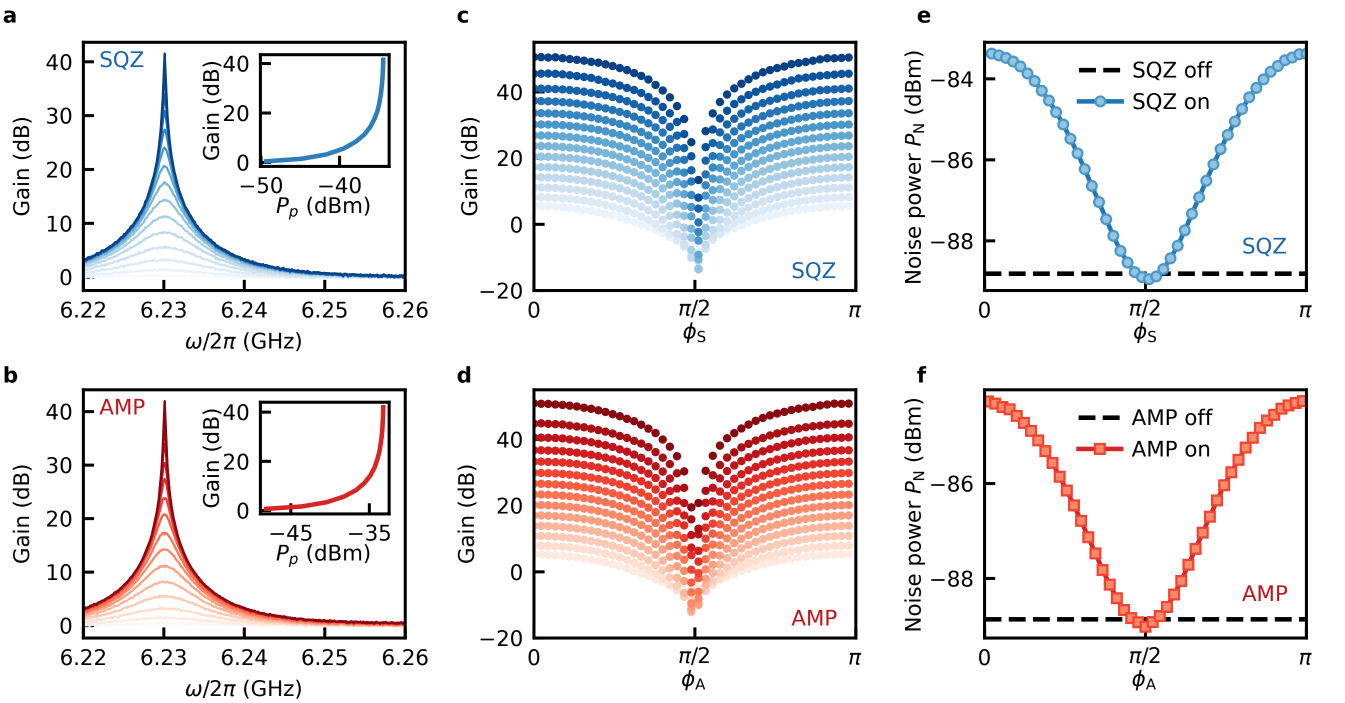

FIG. S9. **Gain and independent vacuum squeezing of the KIPAs. a,b** Phase-insensitive gain of SQZ (**a**) and AMP (**b**) measured as a function of probe frequency $\omega/2\pi$. Increasing the opacity corresponds to increasing the pump power from $P_p = -49$ to -34 dBm for SQZ and from $P_p = -48$ to -33 dBm for AMP. Inset: Extracted maximum gain as a function of P_p. **c,d**, Phase-sensitive gain measured as a function of pump phase. The pump powers match those used in panels a and b. **e,f** The noise power measured along the I-quadrature $P_{\rm N}$ as a function of pump phase. The measurements were taken with only one of SQZ or AMP on at a time. In both panels, the black dashed line corresponds to a measurement taken with both KIPAs off, and corresponds to a reference power level for when vacuum noise is being sent from the MXC plate. Vacuum squeezing occurs for pump phases about $\pi/2$, where the measured power drops below the reference level. For all panels, the devices were tuned to have frequency $\omega_0/2\pi = 6.23$ GHz and the pump frequency was set to $\omega_p/2\pi = 2\omega_0/2\pi = 12.46$ GHz. Panels a,c,e are re-plots of the data presented in Fig. 2 of the main text.

This sets an upper bound for the GBP of $2\pi \times 124$ MHz, which is sufficient to produce 20 dB of vacuum squeezing over 12.4 MHz of bandwidth. Theoretically $I_* \approx 2.38 I_c$ [10]. I_* could be brought closer to this theoretical limit by eliminating weak spots and current-crowding in the resonator [14], which could boost the GBP upper limit by more than a factor of 4 to $2\pi \times 547$ MHz. Extending further, one could consider moving away from resonant geometries to travelling wave designs [15], which has been recently explored in Josephson junction based devices [16].

E. Independent Vacuum Squeezing

In the absence of a probe signal and with the thermal noise source turned off, the field input to SQZ (or AMP when SQZ is off) corresponds to a vacuum state. We make measurements of vacuum squeezing with each amplifier independently by measuring the phase-dependent noise power $P_{\rm N}$ along the I-quadrature when one KIPA is on and the other is off, and comparing it to a measurement where both KIPAs are off (Figs. S9e,f). SQZ and AMP are independently capable of reducing the noise below the reference vacuum level by -0.14 dB and -0.16 dB, respectively. Because these measurements utilize only a single KIPA, the degree of vacuum squeezing that is achieved is limited by noise added by the HEMT amplifier.

F. 1-dB Compression Point

We measure the compression point of both amplifiers by increasing the input signal power signal power $P_{\rm signal}$ while operating the device at a fixed setpoint (frequency, phase, and pump power). The 1-dB compression point

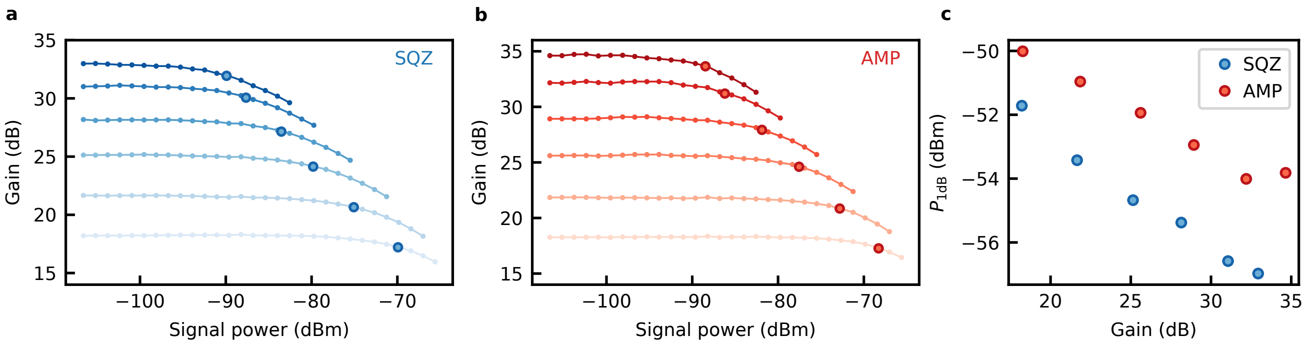

FIG. S10. **1-dB compression point of the KIPAs.** **a**, The SQZ gain measured as a function of input signal power P_{signal}, operated in phase-sensitive mode at maximum amplification ($\phi_{\mathrm{S}} = 0$). The 1-dB compression point in each measurement is indicated by large blue circles. **b**, An equivalent measurement for AMP. **c**, The extracted 1-dB compression points, referred to the output of the devices.

corresponds to the power measured at the output of the amplifier at which the amplifier gain decreases by 1-dB. We perform the measurements in phase-sensitive mode at the settings used in the main text to achieve maximum squeezing ($\omega_{0,S}/2\pi = \omega_{0,A}/2\pi = \omega_0/2\pi = 6.2301$ GHz), with the pump phases set to $\phi_{\mathrm{S}} = \phi_{\mathrm{A}} = 0$ (maximum amplification), Fig. S10a for SQZ and Fig. S10b for AMP. In Fig. S10c we show that both devices achieve substantial 1-dB compression points, which range from -50 dBm to -56 dBm for gains between 20 dB and 35 dB. These values are in close agreement with previous generations of KIPAs [4] and are comparable to the performance of kinetic inductance travelling wave amplifiers [15, 17, 18].

During the squeezing measurements of the main text AMP is operated at a gain of 34 dB. Importantly, to estimate the achieved squeezing it is essential that the AMP gain remains unchanged when SQZ is turned on and off. A concern could be that the noise which is anti-squeezed by SQZ could exceed the 1-dB compression power of AMP, which would cause AMP to compress. This would have the effect of reducing the power at the output of AMP, and therefore overestimate the amount of squeezing. However, from the measurements in Fig. S10b, we observe that when AMP is set to a gain of 34 dB an input signal power greater than -88 dBm is required to saturate the AMP gain. This is 4 orders of magnitude higher than the power of the amplified vacuum state that is transferred from SQZ to AMP. We estimate this to be $P^{S}_{\mathrm{out}} \approx (1/4)G_{\mathrm{S}}\hbar\omega_0 BW$, equal to -129 dBm for $10\log_{10}(G_{\mathrm{S}}) = 18$ dB (the optimal SQZ gain), where BW denotes the SQZ bandwidth, $\hbar$ the reduced Planck constant, ω_0 the resonance frequency of SQZ and 1/4 is the vacuum fluctuations per quadrature.

G. Frequency Dependence of Pump Transmission

In this section we infer the frequency-dependent transmission of the pump power to each device. We first tune each KIPA to a particular frequency ω_0 using the DC current I_{DC}. Next, we measure the phase-insensitive gain for that KIPA as a function of pump power P_p with the pump frequency set to $\omega_p = 2\omega_0$. We then determine the precise pump power required to achieve a phase-insensitive gain of 20 dB, which we define as $P_{p,20\mathrm{dB}}$. Finally, we plot the frequency-dependence of $P_{p,20\mathrm{dB}}$, which we measure across the operational frequency range of each KIPA (Fig. S11).

Both KIPAs show up to 15 dB variations in $P_p(\omega_0)$ over small frequency ranges, which we attribute to reflections in the lines and device at the pump frequency. Despite this, Fig. S11 shows that for both devices $P_{p,20\mathrm{dB}}(\omega_0)$ is reproducible and stable across several thermal cycles of the dilution refrigerator. This demonstrates that the KIPAs have excellent stability and that the pump frequency dependence can be effectively calibrated. For achieving a desired frequency and gain of the KIPAs, we could employ a look-up table that persisted over several months.

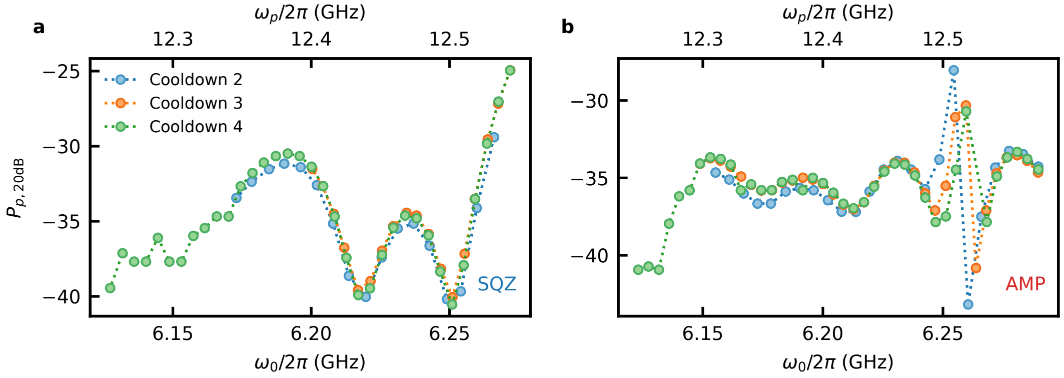

FIG. S11. **Pump power needed for 20 dB of phase-insensitive gain**. **a**, $P_{p,20\mathrm{dB}}$ for SQZ measured as a function of the resonance frequency ω_0. The pump power is plotted for three separate cooldowns. **b**, An equivalent measurement for AMP.

H. Variation of Resonance Frequency and Quality Factors with Magnetic Field

As part of the experiment presented in Figs. 4a-c of the main text, we extract ω_0 and Q_i for SQZ and AMP at zero current ($I_{\mathrm{DC}} = 0$) as a function of the strength of the magnetic field applied parallel to SQZ $B_\parallel$ (Fig. S12). The parameters are extracted from measurements of S_{11}. For all $B_\parallel$, measurements of S_{11} were taken with a signal power of −126 dBm (based on a calibration of the fixed attenuation and line loss using the noise measurements in Section 4 J) referred to the input of the device. We estimate this to result in an average intracavity photon number below one based on the quality factors and applied signal power [19]. Throughout the measurements, the coupling quality factors Q_c of SQZ and AMP were found to remain stable. To keep the net magnetic field aligned in plane with SQZ, each time we increase the field of the primary magnet coil we adjust the field produced by an orthogonal coil which is nominally aligned to be out-of-plane with SQZ, to compensate for any misalignment. We use ω_0 of SQZ as an indicator for the field alignment [20], where ω_0 is maximal for a field aligned along $B_\parallel$.

A gradual decrease in ω_0 for both KIPAs is observed as $B_\parallel$ is increased (Figs. S12a,b). This response was expected for SQZ because magnetic fields are known to increase kinetic inductance by decreasing the supercurrent density [20]. AMP is fixed to the MXC plate well outside of the magnet bore. Nevertheless, the stray magnetic field at its position is expected to be of order 10 mT for $B_\parallel = 2$ T [2]. While the stray field is significantly weaker in magnitude than $B_\parallel$, its alignment is predominantly out-of-plane to AMP and thereby results in a similar shift in ω_0.

The extracted internal quality factors are also found to degrade gradually with $B_\parallel$ (Figs. S12c,d). Interestingly, this effect is more pronounced for AMP than it is for SQZ. This indicates that for the experiments shown in Fig. 4a-c of the main text, the stray field impinging on AMP was likely a key limitation to the degree of squeezing achieved. This is further supported by the fact that we could not proceed beyond $B_\parallel > 2$ T, because the gain of AMP could not be made to exceed 34 dB without the device turning normal.

I. Variation of Resonance Frequency and Quality Factor with Temperature

In Fig. S13 we show ω_0 and Q_i for SQZ, measured as a function of temperature T_H during the experiment presented in Figs. 4d-f of the main text. As in the previous section, these parameters are extracted from measurements of the scattering parameter S_{11}. The monotonic decline in ω_0 with T_H is consistent with the temperature dependence of the kinetic inductance [21]. Correspondingly, we modify I_{DC} for each T_H during the squeezing measurements in Figs. 4d-f to maintain a constant ω_0 throughout the experiment.

J. Amplifier Added Noise

The procedure we use for characterizing the noise added by the KIPAs involves measuring the amplified noise power P_{N} on a single quadrature as a function of the thermal noise source temperature T_{H}, or equivalently the number of thermal photons per quadrature of the input field n_{th}. This procedure is illustrated for AMP in Figs. S14a-d. At each T_{H}, we measure the phase-dependent gain of the KIPA using coherent states (to ensure its operation is stable, Fig. S14a) and the pump-phase-dependent noise power $P_{\mathrm{N}}(\phi_{\mathrm{A}})$ (Fig. S14b). The noise added by the KIPA, stated

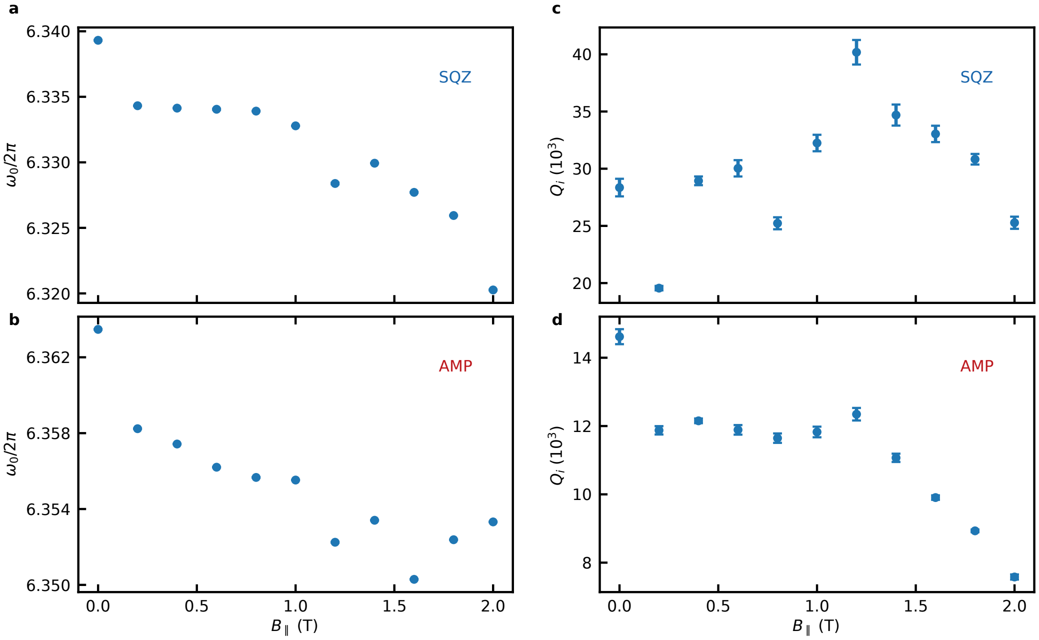

FIG. S12. **Field dependence of KIPAs a,b**, Magnetic field $B_\parallel$ dependence of the resonance frequency $\omega_0/2\pi$ of SQZ (**a**) and AMP (**b**). A gradual decrease in ω_0 is observed for both KIPAs with a 20 MHz decrease for the SQZ and 10 MHz decrease for the AMP at $B_\parallel = 2$ T. **c,d**, Internal quality factor Q_i measured as a function of $B_\parallel$. We observe a decrease in Q_i for both KIPAs, however, AMP is degraded to a lower Q_i (8×10^3) compared to SQZ (20×10^3). Error bars indicate the error on the mean.

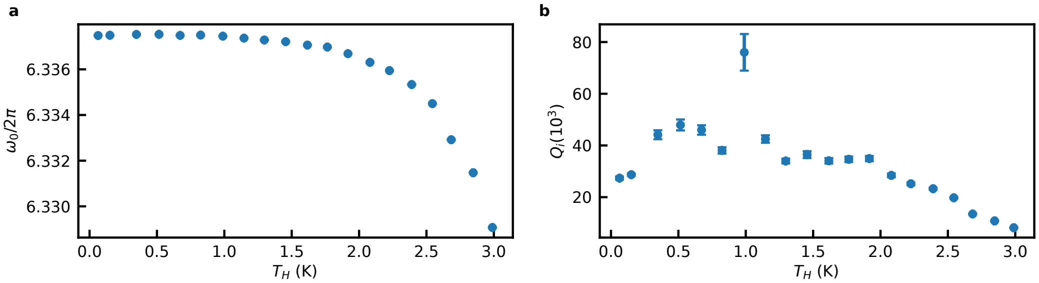

FIG. S13. **Temperature dependence of SQZ a**, The resonance frequency ω_0 declines monotoncially with T_{H}, as expected from the temperature dependence of the kinetic inductance. **b**, Internal quality factor Q_i as a function of temperature. Error bars indicate the error on the mean.

in terms of input-referred photons n_{K}, can then be found from the slope and intercept of the amplified noise power $P_{\mathrm{N}}(n_{\mathrm{th}})$ (Fig. S14c), in accordance with the input-output model (Eqs. 32-33). This also requires that we measure the noise power as a function of n_{th} with both SQZ and AMP deactivated (Fig. S14d), from which we determine that the HEMT adds 6.9 photons of noise to each quadrature. For AMP, the ratio of the slope and y-intercept yields $\frac{1}{\eta_0\eta_1}\left[\frac{1}{4} + n_{\mathrm{A}}\right]$ (Eq. 33). We assume the thermal occupation at the ports of the circulator $n_{\eta 0} = n_{\eta 1} = 0$, which is justified because the circulator is thermalized to the mixing chamber of the dilution refrigerator, which is kept below 44 mK throughout the experiment, and all lines of the dilution refrigerator are heavily attenuated in the relevant frequency band.

We take into account uncertainties in the measured data points and the parameters η_0 and η_1 with a bootstrapping approach. The coordinates of each data point are sampled from a normal distribution around the mean, with a standard deviation given by the propagated measurement error in the n_{th} and P_{N} coordinates. The parameters η_0 and η_1 are sampled from a normal distribution with a 1σ confidence interval ranging from 0.85 to 1, based on the fits and uncertainties in Figs. 3d,e of the main text. The noise figure n_{K} is then determined in $N = 400$ samples by applying a weighted fit through the sampled coordinates, with weights inversely proportional to the data point variance. Taking the mean of the bootstrap samples then gives the estimate for n_{K}.

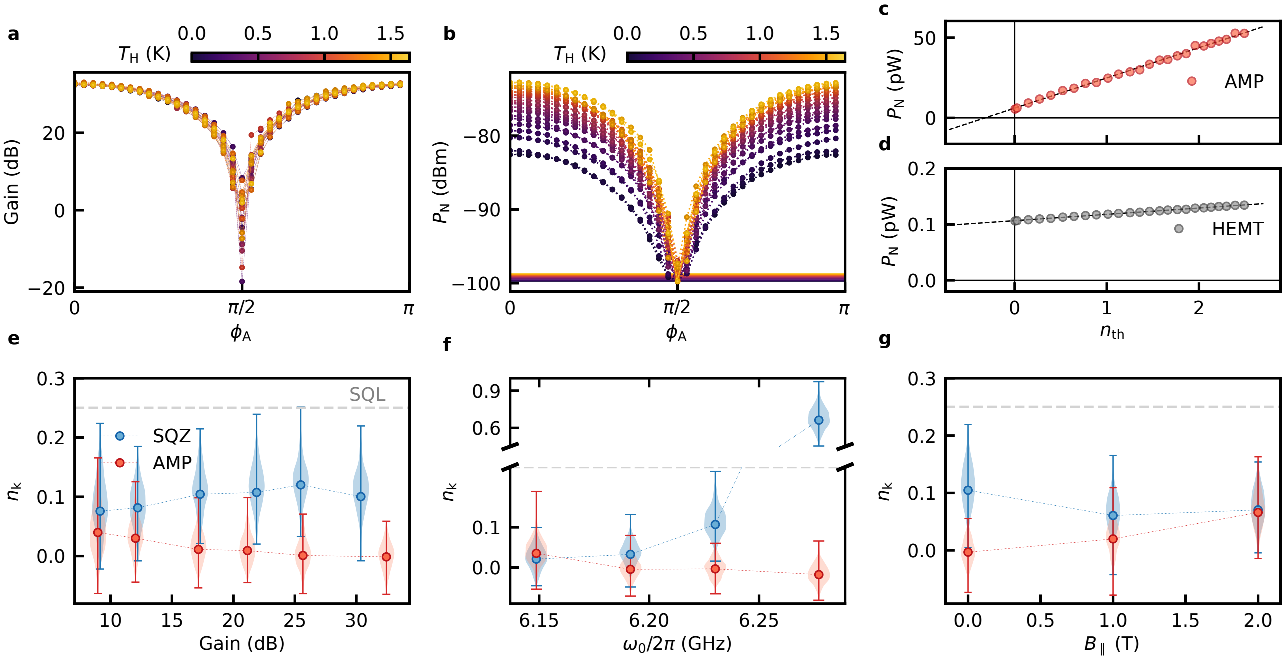

FIG. S14. **Amplifier added noise characterization in anti-squeezing mode.** **a** The phase-dependent gain of AMP, measured for noise source temperatures T_H in the range 60 mK to 1.6 K. Note that the gain is stable and independent of T_H. **b** The corresponding phase-dependent (de-)amplified noise. The output noise P_N increases as the thermal noise source temperature is increased. The colored horizontal lines indicate the output power when AMP is deactivated. We use the data collected at $\phi_A = 0$ for the determination of the added noise in anti-squeezing mode. **c** The noise power P_N vs. number of thermal photons n_{th} (for AMP). The noise added by the amplifier is calculated from the y-intercept and the slope. **d** The noise power vs. n_{th} when both SQZ and AMP are deactivated. Using this measurement we determine the HEMT added noise as $n_H = 6.9$ photons per quadrature. **e** Amplifier added noise vs. gain (at $\omega_0/2\pi = 6.23$ GHz). The horizontal dashed line indicates the standard quantum limit (SQL) of 1/4 photon per quadrature. **f** Amplifier added noise vs. ω_0 (at SQZ Gain = 18 dB and AMP Gain = 34 dB). **g** Amplifier added noise vs. parallel magnetic field $B_\parallel$ (at $\omega_0/2\pi = 6.2301$ GHz, SQZ Gain = 18 dB and AMP Gain = 34 dB).

The amplifier added noise estimate n_K and its probability distribution are plotted in Figs. S14e-g as a function of three parameters: (anti-squeezing) gain, frequency $\omega_0/2\pi$ and parallel magnetic field $B_\parallel$. At the optimal operational gains (34 dB and 18 dB for AMP and SQZ, respectively) used in Fig. 3 of the main text, we estimate the single-quadrature noise figure in anti-squeezing mode to be $n_A^{anti} = 0.00 \pm 0.02$ and $n_S^{anti} = 0.11 \pm 0.02$. It must be noted here that the noise figure of interest for SQZ is in squeezing, instead of anti-squeezing mode. We determine the noise figure in squeezing mode to be $n_S^{sq} = 0.02 \pm 0.02$ by fitting the data in Fig. 4g of the main text with Eq. 36. Such asymmetries in the added noise between squeezing and anti-squeezing mode have been previously documented, in both theory [22] and experiment [23].

5. SUPPLEMENTARY NOTE 5: PUMP CROSSTALK

A. Influence of the SQZ pump on the AMP Gain

In this study, maintaining a consistent AMP gain while activating the SQZ pump is crucial for performing accurate squeezing measurements. To investigate the effect of any potential SQZ pump leakage on the AMP gain, we perform measurements of the AMP gain with the SQZ pump turned on and off. For the measurements where the SQZ pump is activated, the pump power is adjusted to match the squeezing measurements presented in Fig. 3 of the main text ($P_{p,S} = -37$ dBm, corresponding to 18 dB of gain) while keeping $I_{DC} = 0$ for SQZ. In Fig. S15a we present measurements of the AMP gain as a function of ϕ_A for different ϕ_S with a reference measurement taken with the SQZ pump deactivated. In Fig. S15b we zoom into the ϕ_A values corresponding to an AMP gain of 35 dB, close to the optimal operation point of AMP in Fig. 3 of the main text. In Fig. S15c, we show the extracted maximal values of the AMP gain as a function of ϕ_S and compare it to the the maximal AMP gain when SQZ is turned off. We

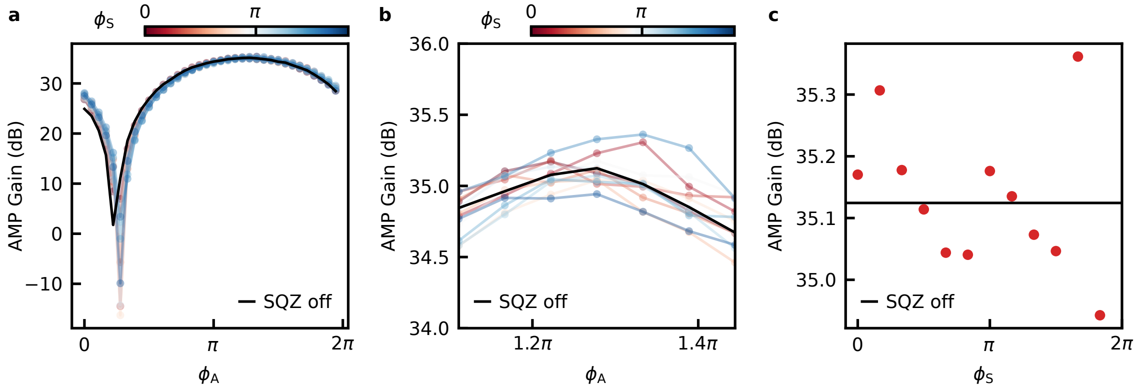

FIG. S15. **AMP gain with SQZ pump on or off measured with a coherent input state.** **a**, AMP gain measured as a function of AMP phase ϕ_A for varying SQZ phase ϕ_S (colorbar) and with SQZ off (black line). **b**, Zoom-in of the region of maximal AMP gain in **a**. **c**, Maximal AMP gain as a function of ϕ_S extracted from **b** compared to the maximal AMP gain measured with SQZ off (black line).

observe no significant trend of the AMP gain with ϕ_S. Furthermore, the mean of the red points (35.13 ± 0.11 dBm) closely matches the AMP gain with the SQZ pump off (35.12 dBm), indicating no significant difference between the two configurations within the margin of error. This provides confidence that the AMP gain is unchanged when the SQZ pump is turned on.

B. Influence of the SQZ Pump on the AMP Amplified Noise and Vice Versa

To further verify that the SQZ pump does not significantly affect the AMP gain, we performed a pump leakage experiment closely resembling the conditions of the squeezing experiments detailed in the main text. While Section 5 A examined the influence on the gain with a coherent tone input, it's important to note that the input in the squeezing experiments is a vacuum state. Therefore, in this section we look at the influence of the SQZ pump on the AMP amplified noise and vice versa.

In the squeezing experiments we tune SQZ and AMP to a mutual resonance frequency ω_0 using DC currents, and supply independent pump tones with a frequency $\omega_p = 2\omega_0$. To investigate the pump crosstalk between SQZ and AMP, we configure both devices as if they were to be used in a squeezing measurement, and then deactivate one device by setting its current to $I_{DC} = 0$. The two pump tones remain on with a mutual frequency of $\omega_p = 2\omega_0$. We then measure the amplified noise power P_N as a function of the pump phases ϕ_S and ϕ_A.

To characterize this crosstalk, we use the configuration where we obtained the largest degree of squeezing (-7.8(2) dB, Fig. 3 of the main text). This occurs at $\omega_0/2\pi = 6.23$ GHz, with pump powers of $P_{p,S} = -37$ dBm (18 dB) and $P_{p,A} = -34$ dBm (34 dB) for SQZ and AMP, respectively.

In Fig. S16a we measure $P_N(\phi_S, \phi_A)$ with SQZ activated and AMP deactivated. We observe no dependence on P_N with ϕ_A, as exemplified by Fig. S16b, which shows the line-cut $P_N(\phi_A)|_{\phi_S=0}$ (for clarity, we plot it as a variation about its mean value). We thus conclude that the pump of AMP has no measurable effect on SQZ. In Fig. S16c we measure $P_N(\phi_S, \phi_A)$ in the reverse configuration, with SQZ deactivated and AMP activated. The line-cut $P_N(\phi_S)|_{\phi_A=0}$ reveals a weak dependence on ϕ_S. We fit the line-cut with a sinusoid and extract an amplitude of 0.15 dB. Whilst this phase-dependent crosstalk does not affect our evaluation of the vacuum noise (which is averaged over all ϕ_S), it may impact our measurement of the squeezed noise level by ± 0.15 dB. We take this into account as an additional systematic uncertainty in our squeezing estimates.

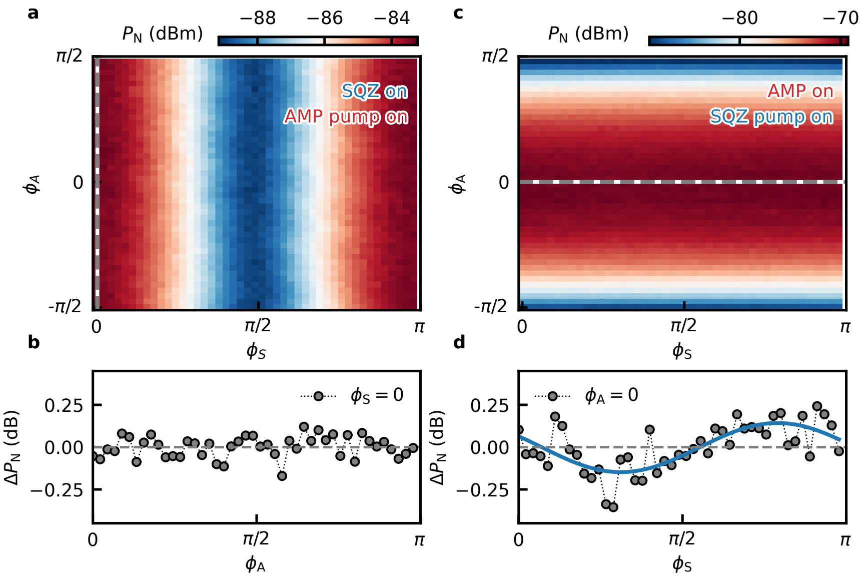

FIG. S16. **Measurement of pump crosstalk between the KIPAs with a vacuum input state. a** Measurement of the influence of the AMP pump on the noise amplification of SQZ. We plot the noise power $P_{\rm N}$ as a function of the pump phases $\phi_{\rm A}$ and $\phi_{\rm S}$ with SQZ activated and AMP tuned out of resonance ($I_{\rm DC} = 0$). The AMP pump power is set at $P_{\rm p,A} = -34$ dBm, the same setting used for optimal squeezing in Fig. 3 of the main text. **b** A linecut at $\phi_{\rm S} = 0$ of $P_{\rm N}$, plotted as a variation about its mean value. We observe no dependence of $P_{\rm N}$ with $\phi_{\rm A}$. **c** Measurement of the influence of the SQZ pump on the noise amplification of AMP. The measurement is the same as in panel **a**, with the roles of SQZ and AMP reversed. **d** A linecut of $P_{\rm N}$ at $\phi_{\rm A} = 0$, plotted as a variation about its mean, reveals a small sinusoidal dependence on $\phi_{\rm S}$ with an amplitude of 0.15 dB.

6. SUPPLEMENTARY NOTE 6: SUPPLEMENTARY FIGURES

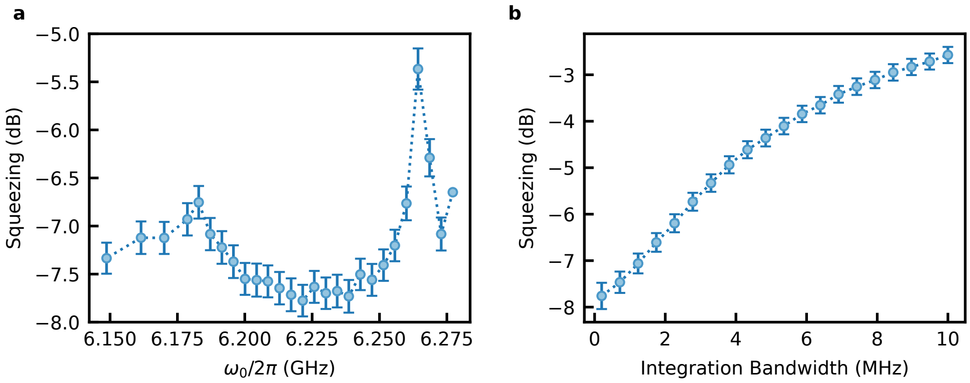

FIG. S17. **Frequency and integration bandwidth dependence of squeezing. a**, Squeezing as a function of the resonance frequency ω_0 of the KIPAs. We demonstrate squeezing over a range of approximately 150 MHz by varying $I_{\rm DC}$ and aligning ω_0 of both SQZ and AMP. For each data point, the gain of SQZ is optimized to reach the maximum squeezing level. The gain of AMP is stabilized at 34 dB. We achieve maximum vacuum squeezing at $\omega_0/2\pi = 6.23$ GHz, corresponding to $I_{\rm DC} = 0.90$ mA for SQZ and $I_{\rm DC} = 0.97$ mA for AMP. The frequency range is limited by the critical currents of the superconducting films. **b**, Squeezing as a function of the integration bandwidth at the optimal operation point of Fig. 3c of the main text. Error bars indicate the error on the mean.

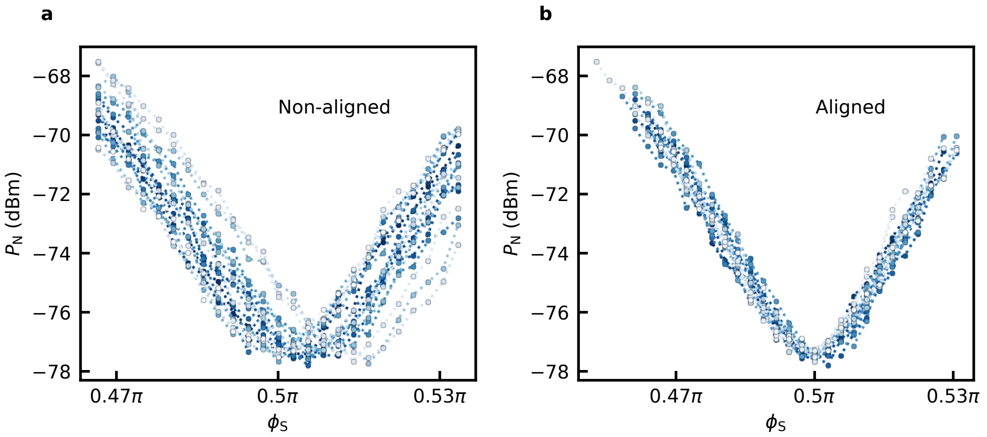

FIG. S18. **Alignment procedure for the squeezing measurements, accounting for slow phase drifts.** **a**, Raw measured data of the noise power P_{N} for Fig. 3c of the main text. The P_{N} reaches approximately the same minimum for each measurement repetition, with a minor variation in the pump phase. **b**, The raw data is aligned by fitting each repetition with a parabolic function and centring the minima on $\phi_{\mathrm{S}} = \pi/2$.

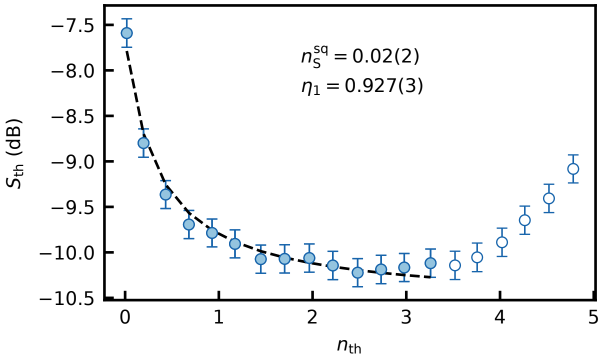

FIG. S19. Fit of thermal squeezing vs. number of thermal noise photons n_{th} to the thermal squeezing model in Supplementary Eq. S36. The fit result yields the amplifier added noise $n_{\mathrm{S}}^{\mathrm{sq}}$ and insertion loss η_1 for the setup displayed in Fig. 4d of the main text. Error bars indicate the error on the mean.

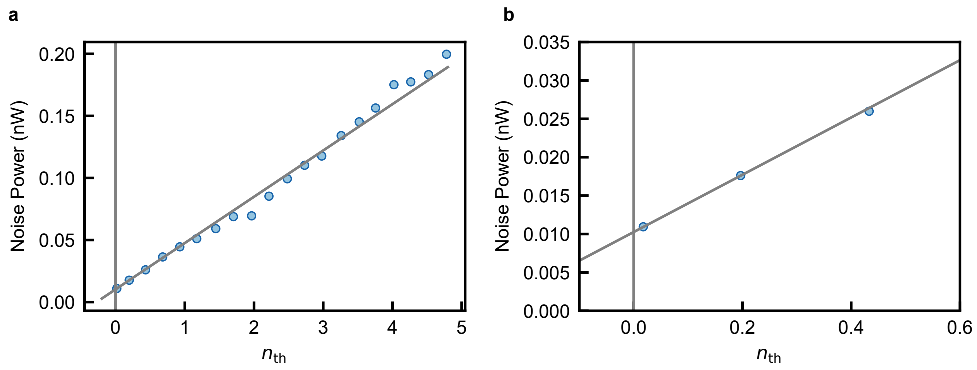

FIG. S20. **Noise extrapolation to $n_{\mathrm{th}} = 0$ for determining the vacuum level in the thermal squeezing measurements.** Due to the increased heat load of the modified setup depicted in Fig. 4d of the main text, the minimum T_{H} we could reach was 80 mK, as opposed to 10 mK in the regular squeezing measurements. Therefore, we obtain the vacuum level by extrapolating $P_{\mathrm{N}}^{\mathrm{A}}$ to $T_{\mathrm{H}} = 0$ K. **a**, Noise power vs. thermal noise photons per quadrature n_{th}. In this measurement, both SQZ and AMP are turned off. We observe a linear increase in the noise power as a function of the number of thermal noise photons. **b**, Zoom-in of panel **a**. We extrapolate a linear fit back to $n_{\mathrm{th}} = 0$ to obtain the vacuum noise reference of 0.1025 nW, or $P_{\mathrm{N}}^{\mathrm{vac}} = -79.9$ dBm. The extrapolated $P_{\mathrm{N}}^{\mathrm{vac}}$ is slightly lower than the first measured value at $T = 80$ mK ($n_{\mathrm{th}} = 0.017$, $P_{\mathrm{N}} = -79.61$ dBm), and thus prevents overestimating the squeezing level in Fig. 4f of the main text.

[1] W. Vine, A. Kringhøj, M. Savytskyi, D. Parker, T. Schenkel, B. C. Johnson, J. C. McCallum, A. Morello, and J. J. Pla, Latched detection of zeptojoule spin echoes with a kinetic inductance parametric oscillator, Sci. Adv. **10**, eadm7624 (2024).

[2] Bluefors Oy AMI 6-1-1 T Integrated cryogen-free superconducting magnet system - magnet specifications and performance sheet 15632 (2022).

[3] S. Simbierowicz, V. Vesterinen, J. Milem, A. Lintunen, M. Oksanen, L. Roschier, L. Grönberg, J. Hassel, D. Gunnarsson, and R. E. Lake, Characterizing cryogenic amplifiers with a matched temperature-variable noise source, Rev. Sci. Instrum. **92**, 034708 (2021).

[4] D. J. Parker, M. Savytskyi, W. Vine, A. Laucht, T. Duty, A. Morello, A. L. Grimsmo, and J. J. Pla, Degenerate Parametric Amplification via Three-Wave Mixing Using Kinetic Inductance, Phys. Rev. Appl. **17**, 034064 (2022).

[5] D. M. Pozar, *Microwave Engineering:* 4th *edition* (Wiley, Hoboken, NJ, 2012).

[6] S. Chaudhuri, D. Li, K. D. Irwin, C. Bockstiegel, J. Hubmayr, J. N. Ullom, M. R. Vissers, and J. Gao, Broadband parametric amplifiers based on nonlinear kinetic inductance artificial transmission lines, Appl. Phys. Lett. **110**, 152601 (2017).

[7] N. T. Bronn, Y. Liu, J. B. Hertzberg, A. D. Córcoles, A. A. Houck, J. M. Gambetta, and J. M. Chow, Broadband filters for abatement of spontaneous emission in circuit quantum electrodynamics, Appl. Phys. Lett. **107**, 172601 (2015).

[8] D. Bothner, M. Knufinke, H. Hattermann, R. Wölbing, B. Ferdinand, P. Weiss, S. Bernon, J. Fortágh, D. Koelle, and R. Kleiner, Inductively coupled superconducting half wavelength resonators as persistent current traps for ultracold atoms, New J. Phys. **15**, 093024 (2013).

[9] S. Probst, F. B. Song, P. A. Bushev, A. V. Ustinov, and M. Weides, Efficient and robust analysis of complex scattering data under noise in microwave resonators, Rev. Sci. Instrum. **86**, 024706 (2015).

[10] J. Zmuidzinas, Superconducting Microresonators: Physics and Applications, Annu. Rev. Condens. Matter Phys. **3**, 169 (2012).

[11] X. Zhou, V. Schmitt, P. Bertet, D. Vion, W. Wustmann, V. Shumeiko, and D. Esteve, High-gain weakly nonlinear flux-modulated Josephson parametric amplifier using a SQUID array, Phys. Rev. B **89**, 214517 (2014).

[12] S. Boutin, D. M. Toyli, A. V. Venkatramani, A. W. Eddins, I. Siddiqi, and A. Blais, Effect of Higher-Order Nonlinearities on Amplification and Squeezing in Josephson Parametric Amplifiers, Phys. Rev. Appl. **8**, 054030 (2017).

[13] M. Xu, R. Cheng, Y. Wu, G. Liu, and H. X. Tang, Magnetic Field-Resilient Quantum-Limited Parametric Amplifier, PRX Quantum **4**, 010322 (2023).

[14] J. R. Clem and K. K. Berggren, Geometry-dependent critical currents in superconducting nanocircuits, Phys. Rev. B **84**, 174510 (2011).

[15] M. Malnou, M. Vissers, J. Wheeler, J. Aumentado, J. Hubmayr, J. Ullom, and J. Gao, Three-Wave Mixing Kinetic Inductance Traveling-Wave Amplifier with Near-Quantum-Limited Noise Performance, PRX Quantum **2**, 010302 (2021).

[16] J. Y. Qiu, A. Grimsmo, K. Peng, B. Kannan, B. Lienhard, Y. Sung, P. Krantz, V. Bolkhovsky, G. Calusine, D. Kim, *et al.*, Broadband squeezed microwaves and amplification with a josephson travelling-wave parametric amplifier, Nat. Phys. **19**, 706–713 (2023).

[17] M. R. Vissers, R. P. Erickson, H. Ku, L. Vale, X. Wu, G. C. Hilton, and D. P. Pappas, Low-noise kinetic inductance traveling-wave amplifier using three-wave mixing, Appl. Phys. Lett. **108**, 012601 (2016).

[18] B. Ho Eom, P. K. Day, H. G. Leduc, and J. Zmuidzinas, A wideband, low-noise superconducting amplifier with high dynamic range, Nat. Phys. **8**, 623 (2012).

[19] A. Bruno, G. De Lange, S. Asaad, K. L. Van Der Enden, N. K. Langford, and L. Dicarlo, Reducing intrinsic loss in superconducting resonators by surface treatment and deep etching of silicon substrates, Appl. Phys. Lett. **106**, 182601 (2015).

[20] J. E. Healey, T. Lindström, M. S. Colclough, C. M. Muirhead, and A. Y. Tzalenchuk, Magnetic field tuning of coplanar waveguide resonators, Appl. Phys. Lett. **93**, 043513 (2008).

[21] A. J. Annunziata, D. F. Santavicca, L. Frunzio, G. Catelani, M. J. Rooks, A. Frydman, and D. E. Prober, Tunable superconducting nanoinductors, Nanotechnology **21**, 445202 (2010).

[22] C. M. Caves, Quantum limits on noise in linear amplifiers, Phys. Rev. D **26**, 1817 (1982).

[23] L. Zhong, E. Menzel, R. Di Candia, P. Eder, M. Ihmig, A. Baust, M. Haeberlein, E. Hoffmann, K. Inomata, T. Yamamoto, *et al.*, Squeezing with a flux-driven Josephson parametric amplifier, New J. Phys. **15**, 125013 (2013).
